# Ammonium *N*-(pyridin-2-ylmethyl)oxamate (AmPicOxam): A Novel Precursor of Calcium Oxalate Coating for Carbonate Stone Substrates

**DOI:** 10.3390/molecules28155768

**Published:** 2023-07-30

**Authors:** Anna Pintus, M. Carla Aragoni, Gianfranco Carcangiu, Veronica Caria, Simon J. Coles, Eleanor Dodd, Laura Giacopetti, Domingo Gimeno, Vito Lippolis, Paola Meloni, Simone Murgia, Antonia Navarro Ezquerra, Enrico Podda, Claudia Urru, Massimiliano Arca

**Affiliations:** 1 Dipartimento di Scienze Chimiche e Geologiche, Università degli Studi di Cagliari, S. S. 554 Bivio per Sestu, 09042 Monserrato, CA, Italy; apintus@unica.it (A.P.); aragoni@unica.it (M.C.A.); veronica.caria@yahoo.it (V.C.); lippolis@unica.it (V.L.); s.murgia.chem@gmail.com (S.M.); enrico.podda@unica.it (E.P.); claudia.urru01@universitadipavia.it (C.U.); 2 Consiglio Nazionale Delle Ricerche (CNR), Istituto di Scienze dell’Atmosfera e Del Clima (ISAC), UOS di Cagliari c/o Dipartimento di Fisica, Università degli Studi di Cagliari, S. S. 554 Bivio per Sestu, 09042 Monserrato, CA, Italy; g.carcangiu@isac.cnr.it; 3 National Crystallography Service, School of Chemistry, University of Southampton, Southampton SO17 1BJ, UK; s.j.coles@soton.ac.uk (S.J.C.); eleanor.dodd@soton.ac.uk (E.D.); 4 Facultat de Ciències de la Terra, Universitat de Barcelona, c/Martí i Franquès s/n, 08028 Barcelona, Spain; d.gimeno.torrente@gmail.com; 5 Dipartimento di Ingegneria Meccanica, Chimica e dei Materiali, Via Marengo 2, 09123 Cagliari, CA, Italy; paola.meloni@unica.it; 6 Laboratorio Colle di Bonaria, Università degli Studi di Cagliari, Via Ravenna snc, 09125 Cagliari, CA, Italy; 7 Departamento de Tecnología de la Arquitectura, EPSEB-UPC, Avda. Doctor Marañón, 44-50, 08028 Barcelona, Spain; antonia.navarro@upc.edu; 8 Centro Servizi di Ateneo per la Ricerca (CeSAR), Università degli Studi di Cagliari, S. S. 554 Bivio Sestu, 09042 Monserrato, CA, Italy

**Keywords:** calcite, oxamate, marble, limestone, conservation, cultural heritage, DFT

## Abstract

Ammonium *N*-(pyridin-2-ylmethyl)oxamate (AmPicOxam), synthesized from *O*-methyl-*N*-(pyridin-2-ylmethyl)oxamate, was spectroscopically and structurally characterized and assayed as a novel precursor for the protection and consolidation of carbonate stone substrates. An in-depth characterization of treated and untreated biomicritic limestone and white Carrara marble samples was carried out by means of SEM microscopy, X-ray powder diffraction, helium pycnometry, determination of water transport properties, and pull-off tests. The improved solubility (1.00 M, 16.5% *w*/*w*) of the title compound with respect to ammonium oxalate (0.4 M, 5% *w*/*w*) results in the formation of a thicker protective coating of calcium oxalate (CaOx) dihydrate (weddellite) on marble and biomicrite samples after the treatment with 5% and 12% *w*/*w* water solutions, producing a reduction in the stone porosity and increased cohesion. Theoretical calculations were carried out at the DFT level to investigate both the electronic structure of the *N*-(pyridin-2-ylmethyl)oxamate anion and the hydrolysis reaction leading from AmPicOxam to CaOx.

## 1. Introduction

Natural carbonate stones, such as marble and limestone, have accompanied the development of humankind through history as election materials in architecture and figurative art [1,2]. These materials are susceptible to physical, chemical, and biological weathering processes [3]. From a physical point of view, calcite undergoes spontaneous weakening and development of a network of microcracks [4,5] due to its anisotropic thermal behavior during day/night and heating/cooling cycles [6]. Another major cause of degradation is represented by the dissolution of calcite in water [7,8] (*K*_sp_ = 3.27 × 10^−9^ at 25 °C) [9], accelerated by the acidity of rain due to anthropogenic activities, resulting in the replacement of calcium carbonate by soluble minerals, such as gypsum and calcium nitrate [10,11]. In addition, lichens, fungi, algae, and several types of lithoautotrophic bacteria can produce acidic metabolites that further contribute to the acidic dissolution of the stones [12,13,14]. All these phenomena contribute to the weathering processes that represent a major problem in the conservation not only of the artistic details carved in stone but also of the architectural elements in buildings or even the mortars used for their construction.

The role played by chemistry has always been crucial in developing new materials for the protection and restoration of stone artifacts of archaeological and, in general, cultural heritage interest. Consolidating agents can penetrate the network of cracks in weathered stone and either precipitate or polymerize in situ [15]. The consolidation restores the adhesion between the stone grains and limits further degradation and the subsequent macroscopic effects, such as roughness of the stone surfaces and material loss [16].

To date, only a limited number of classes of inorganic compounds have been tested as conservation agents for carbonate stone substrates [17], including alkaline earth hydroxides [18,19], re-mineralizing calcium carbonate [20], or salts such as diammonium hydrogen phosphate (NH_4_)_2_(HPO_4_) [10,21,22]. In recent decades, a renewed interest has been focused on the use of ammonium oxalate (NH_4_)_2_(C_2_O_4_) (AmOx) as a passivating and protective agent [23,24,25,26,27], whose reaction with calcium carbonate results in the formation of calcium oxalate (CaOx) hydrate Ca(C_2_O_4_)·*n*H_2_O (*n* = 1; whewellite, *K*_sp_ = 2.0 × 10^−9^; *n* = 2, weddellite, *K*_sp_ = 3.8 × 10^−9^ at 25 °C; Equation (1)) [28,29,30,31,32,33]:

CaCO_3(s)_ + (NH_4_)_2_(C_2_O_4_)_(aq)_ + (*n* – 1)H_2_O_(l)_ → Ca(C_2_O_4_)·*n*H_2_O_(s)_ + 2 NH_3(g)_ + CO_2(g)_ (1)

More rarely, trihydrate Ca(C_2_O_4_)·3H_2_O (caoxite) has been observed [34,35]. It is worth noting that the formation of a CaOx patina ultimately mimics the action of natural microorganisms, which generate biologically induced constructive carbonate–oxalate patinas (both in natural rock and architectural stained-glass windows) [36,37,38] containing both whewellite and weddellite [39], especially in non-contaminated environments where the formation of sulphates on the patinas is prevented [40]. Furthermore, AmOx converts gypsum deposits, resulting from the action of acid rain, to whewellite (Equation (2)) [17]:

CaSO_4_·2H_2_O_(s)_ + (NH_4_)_2_(C_2_O_4_)_(aq)_ → Ca(C_2_O_4_)·H_2_O_(s)_ + (NH_4_)_2_SO_4(aq)_ + H_2_O_(l)_ (2)

As CaOx hydrate features a low solubility and is stable under acidic conditions, the precipitation and formation of a coating of whewellite/weddellite is a powerful tool for consolidating and protecting weathered carbonate rocks. This notwithstanding, the penetration depth of the AmOx treatment into the stone network is limited, due to its poor solubility in water at room temperature. Therefore, the treatments are carried out by using diluted solutions (maximum 0.4 M, 5% *w*/*w*) or even dispersions (for example in the poultice applications) [41]. Such disadvantages may be avoided by replacing the oxalate anion with related derivatives that have a higher water solubility and are therefore able to precipitate as insoluble calcium salts on the stone material or to generate the oxalate anion by hydrolysis [41,42,43,44,45,46]. In this context, dimethyloxalate [42] and diethyloxalate [35] have been tested as precursors of the oxalate anion. In particular, treatments with dimethyloxalate, which is sensibly more soluble in water than the ethyl homologue, have been recently described in a sequential treatment including AmOx and calcium acetate [42]. Ammonium salts of oxalic acid monoesters are particularly prone to hydrolysis but have proven to be promising precursors for the generation of whewellite protective layers due to their greater penetration capability [32,43]. In all cases, the thickness of the CaOx coating depends on the nature of the stone samples. The thickness of the deposition layer on marble samples ranges between 10 and 50 μm, while a larger variability was found when more porous limestones were treated, largely depending on the nature of the treated sample [41,43,44]. Contrary to oxalic esters, monoamides (oxamic acid derivatives) display a larger water stability [45] and can generate calcium oxamate coatings, although they can undergo hydrolysis over a longer time scale [46]. In recent years, the authors have reported on the use of ammonium monoalkyloxalate salts (NH_4_)(ROC(O)COO) (R = Me and Et; AmMeOx and AmEtOx, respectively) as useful precursors of CaOx [32,33], while ammonium oxamate (NH_4_)(NH_2_C(O)COO) [32] (AmOxam) and *N*-phenyloxamate (NH_4_)(PhNHC(O)COO) [35] (AmPhOxam) were successfully tested for the conservative treatment of white Carrara marble and biomicritic limestone.

Notably, the introduction of an aromatic substituent at the N-atom of the oxamate anion in AmPhOxam resulted in a dramatic decrease in water solubility as compared to AmOxam [46].

**Scheme 1:**
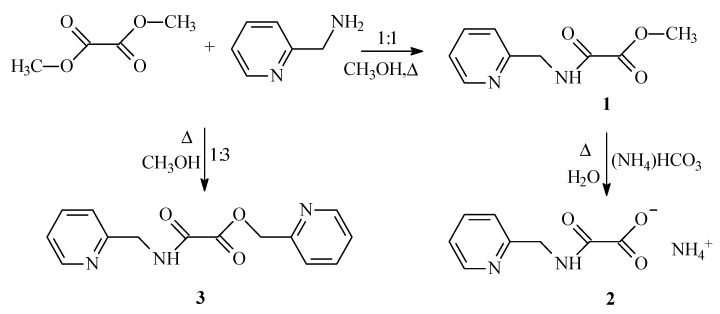
Synthesis of compounds **1**–**3**.

Following the results of the search for oxalic acid derivatives as consolidants, we have turned to the introduction of a basic N-heterocyclic substituent, namely pyridin-2-ylmethyl (2-picolyl), which can potentially increase water solubility and simultaneously neutralize the acidity of the ammonium cation (pH = 3.75 for 5% *w*/*w* AmOx aqueous solution) [32]. We report here on the consolidation ability of ammonium *N*-(pyridin-2-ylmethyl)oxamate (AmPicOxam, compound **2** in Scheme 1) towards white Carrara marble and biomicrite limestone.

## 2. Results and Discussion

### 2.1. Synthesis and Characterization

The number of reported ammonium monosubstituted oxamic acid derivatives, (R′_4_N)(RNHC(O)COO) (R, R′ = H, alkyl, or aryl substituents) is limited, and the structural data of less than 15 compounds have been deposited at the Cambridge Structural Database (CSD) to date [47]. In most cases, the protonation of the amine RNH_2_ used for the synthesis also provides the cation RNH_3_^+^ [48,49,50,51,52]. To synthesize ammonium *N*-(pyridin-2-ylmethyl)oxamate (AmPicOxam), dimethyloxalate was made to react with 2-picolylamine in 1:1 molar ratio. The resulting monoester (*O*-methyl-*N*-(pyridin-2-ylmethyl)oxamate, **1**) was subsequently converted to AmPicOxam (**2**) by reaction with (NH_4_)HCO_3_ in water, thanks to the larger tendency toward hydrolysis of the monoester compared to the monoamide in oxalic acid derivatives (Scheme 1) [34]. Notably, when compound **1** was reacted with an excess of 2-picolylamine, the corresponding *N*,*N*′-bis(pyridin-2-ylmethyl)oxalamide (**3**) was directly obtained. Microanalytical and ^1^H-NMR spectroscopic measurements showed that small molar amounts of compound **3** were also found during the synthesis of compound **1**, which was therefore purified by column chromatography.

All compounds were fully characterized by microanalytical (m.p. determination, elemental analysis), diffractometric (Figure 1 and Figures S1–S3, Tables S1–S4), and spectroscopic (^1^H-NMR, FT-IR, UV-Vis) techniques (Figures S4–S14). The solubility of compound **2** (16.5% *w*/*w*, corresponding to a 1.0 M concentration for a saturated solution; *K*_sp_ = 1.0) was determined spectrophotometrically, after recording a calibration curve (Figure S10).

Single crystals suitable for an X-ray diffraction analysis were grown by slow infusion of diethylether into a dichloromethane solution of compound **1** (Figures S1 and S2, Tables S1 and S2). The crystal structure revealed that compound **1** adopts a quasi-planar structure (O2–C1–C2–O3 = 169.5(1)°), with the only exception of the pyridin-2-ylmethyl substituent, with the two carbonyl group disposed in an antiperiplanar conformation (Figure S1). Notably, pairs of anions interact to each other in a head-to-tail fashion via N–H···N hydrogen bonds (HB) involving the N–H groups of one unit and the pyridine N-atom of the other (N1···N2^i^, 2.932(1) Å; H1···N2^i^, 2.104(1) Å; N1–H1···N2^i^, 163.4(1)°; ^i^ = 1 − x, 1 − y, 1 − z; Figure S2). Weak C–H···O contacts determine the crystal packing of the dimeric units (C5^ii^···O1, 3.385(2) Å, C8^iii^···O2, 3.453(2) Å; C7^iv^ ···O2, 3.292(1) Å; ^ii^ = 1 − x, −1/2 + y, 3/2 − z; ^iii^ = −x, 1 − y, 1 − z, ^iv^ = x, −1 + y, z).

**Figure 1:**
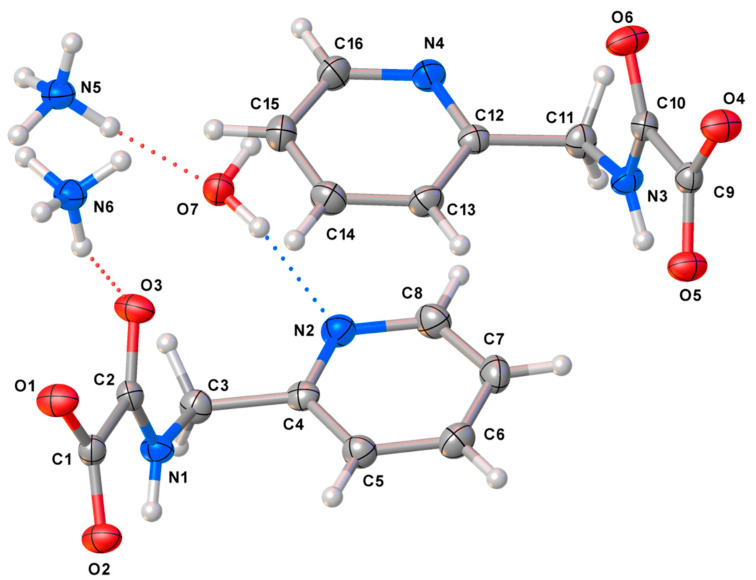
Molecular structure and atom numbering scheme for **2**·1/2H_2_O. Thermal ellipsoids were drawn at a 50% probability level.

Crystals of compound **2**·1/2H_2_O suitable for an X-ray diffraction analysis were grown by slow evaporation of a DMSO solution. The asymmetric unit comprises two crystallographically independent units of both the anion and the cation and one co-crystallized water molecule (Figure 1 and Figure S3, Tables S1 and S3).

The molecular units of the anions show a periplanar conformation (average O–C–C–O torsion angle 168.8(1)°), contrary to what was observed for compound **1**. The two PicOxam^−^ anions show a head-to-tail disposition, allowing for a slipped face-to-face orientation of the two parallel pyridine rings, with the corresponding centroids, not shown in Figure 1, having a distance of 3.90 Å, suggesting a weak π-π interaction of the same order as those recently reported for different supramolecular assemblies [53]. The crystal packing features a complex network of HB interactions, which can be summarized as follows (Table S4 and Figure S3): (i) the co-crystallized water molecule bridges two pyridyl moieties of two PicOxam^−^ anions and two ammonium cations through O–H···N and N–H···O interactions, respectively (Table S4, Figure S3a); (ii) the ammonium cations are engaged in N–H···O interactions with PicOxam^−^ anions (Table S4, Figure S3b,c); (iii) PicOxam^−^ anions are coupled into dimeric $R_{2}^{2}(10)$ motifs (Table S4, Figure S3d).

Finally, single crystals of compound **3** were grown by slow infusion of n-hexane into a dichloromethane solution. An X-ray diffraction analysis confirmed the nature of the compound, whose crystal structure was previously reported (CSD code POWGEQ) [54].

### 2.2. Reaction of Compound ***2*** with CaCO_3_

**Scheme 2:**
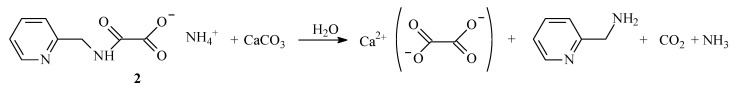
Reaction of compound **2** with CaCO_3_ in water.

The reactivity of stoichiometric amounts of compound **2** (4% *w*/*w*) with pure CaCO_3_ powder was investigated in water. After 24 h at r.t., the insoluble fraction was collected and analyzed by FT-IR spectroscopy and powder X-ray diffraction (PXRD). The diffractogram (Figure S15) clearly showed two different crystalline phases attributed to calcite and weddellite, respectively, and the absence of any additional peaks attributable to residual calcium *N*-(pyridin-2-ylmethyl)oxamate. Therefore, it was hypothesized that the hydrolysis reaction of compound **2** shown in Scheme 2 would occur in the presence of calcium carbonate.

### 2.3. Carbonate Stone Samples

The stone samples belong to two different lithotypes, namely white Carrara marble and biomicrite limestone. The marble samples have a good homogeneity, characterized by a granoblastic texture resulting in a mosaic or a non-oriented polygonal fabric, with a grain size in the range of 100–150 µm. The subhedral CaCO_3_ crystals, with well-defined straight boundaries, often showed 120° junctions. In addition to calcite, which accounts for more than 99% of the stone substrate, small amounts of muscovite/illite and other phyllosilicates, as well as opaque minerals, are included in the marble texture (Table 1 and Table S5). The limestone samples consist of biomicrite (Folk classification), with a remarkable textural and compositional variability in a vertical profile and exhibit poor physical–mechanical properties. In fact, the face of the quarry has a frontal cut some dozens of meters thick, and therefore there are several degrees of variation in the rock characteristics, with a transition to marly rocks at the bottom of the succession. If we consider a textural classification more suitable in terms of decay, there is a range between mudstone–wackestone (and locally even packstone), locally very rich in nektoplanctonic thin-walled forms. The carbonate sediment appears to have undergone early diagenesis, since the internal cavities have not been collapsed by compaction and are currently mostly filled with drusy sparitic calcite. Some terms look richer in detritic fragments, including for the allochems thick-walled forams, coralline debris, algae, etc., as well as an increase in quartz, mica, and other silicate fragments. Some beds are constituted mainly of peloids aggregated by calcite rim cement (grainstone–packstone) and show considerable interparticular sedimentary-early diagenetic (i.e., in a broad sense, primary) porosity. Both these later explained beds and those with less infilling of the foram cameras with drusy sparite cement are a priori those with a predictably worse behavior during weathering of the rock. Exfoliation, alveolation, erosion, pulverization, and crumbling are the most common weathering-induced decay forms. Among secondary components, very small (62–125 µm) grains of monocrystalline quartz (3.5%) and possibly iron oxyhydroxides or oxidized glauconite could be identified.

Stone samples, cut in prisms of about 2.0 × 2.0 × 8.0 cm or cubes of 2.0 × 2.0 × 2.0 cm, were characterized by colorimetry, ultrasonic pulse velocity measurements, helium pycnometry, and surface roughness, in the case of marble before and after artificial thermal weathering, consisting of a steep heating (from r.t. to 500 °C, 8.4 °C/min), a static heating (500 °C, 7 h), and a slow cooling to r.t. (3 °C/min; Figure S16).

In weathered marble samples, the texture revealed microcracks around the edges of specimens and a remarkable detachment of boundary grains (Figure S17). The weathering process results in a general degradation of the dynamic and structural bulk and surface properties with respect to those of the “intact” samples (Table S5). Indeed, the characterization of weathered samples highlighted a microstructure strictly recalling that exhibited by naturally weathered marble [55], thus confirming the appropriateness of the adopted artificial weathering method.

Stone samples were treated by static immersion in aqueous solutions of compound **2** according to the procedure previously reported for related oxalate and oxamate derivatives [32,33,34,35]. The effects of the treatments were investigated by using solutions with concentrations 5.0% and 12.0% *w*/*w* (0.25 and 0.61 M, respectively) on both types of lithotypes. Both solutions display pH values of about 6.5, and conductivity values of 8.80 ± 0.04 and 28.8 ± 0.1 mS/cm, for 5% and 12% *w*/*w* solutions, respectively (Table S6).

### 2.4. Treatment of Carrara Marble Samples

Macroscopic observation of the treated marble samples did not show any evident chromatic alteration (see below). Under observation with optical microscopy (Figure S18), an apparent reduction in grain detachment was observed locally in the sample treated with the 5% *w*/*w* solution. In the case of the samples treated with the most concentrated solution, newly formed layers irregularly distributed on the surface could be observed.

**Figure 2:**
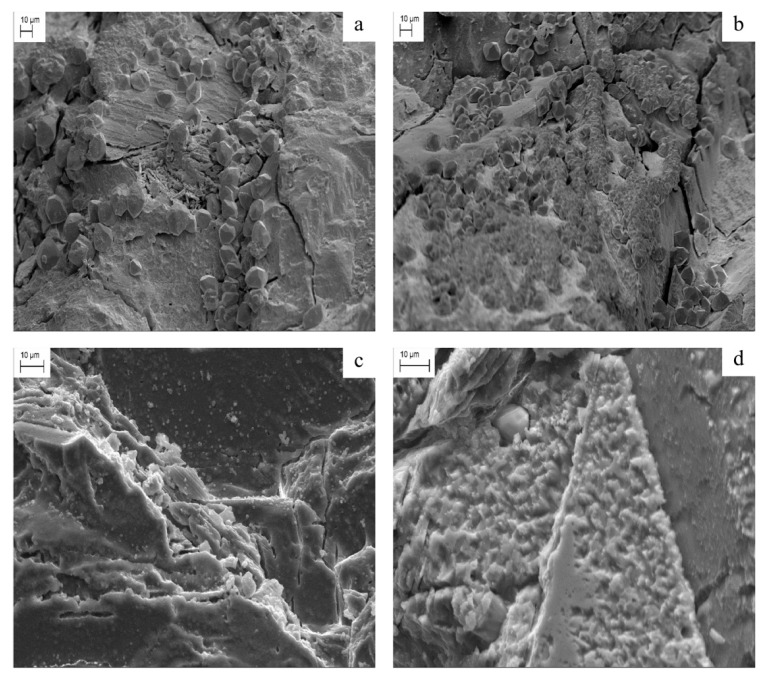
SEM images of weathered Carrara marble treated with a 5% (**a**,**b**) and 12% *w*/*w* (**c**,**d**) solution of compound **2**.

SEM images of the weathered marble samples treated with the 5% *w*/*w* solution showed larger crystals (average size 20 µm) distributed over the surfaces and localized on the fractures and grain boundaries (Figure 2a,b). Where the crystals are more closely assembled, they coalesce to form coatings. In addition to the larger crystals, a second type of deposition derives from extremely minute crystals (< 2 µm), which form a more uniform protective layer on the surface. A section of the coating is shown in Figure S19.

When treated with the 12% *w*/*w* solution of compound **2**, the surface showed several very small crystals (~1 µm) that, as in the previous case, were preferentially concentrated at cracks and grain boundaries, thus forming localized coatings (Figure 2c,d).

In order to investigate the nature of the crystal deposition on the stone surface, PXRD analyses were carried out on both a powdered portion of the treated surface and directly on the surface of bulk samples. In all cases, in agreement with what was observed after reaction with CaCO_3_ powder, the PXRD patterns allowed us to clearly identify weddellite as the only component of the coating formed by treatment with the 5% and 12% *w*/*w* solutions (Figures S20 and S21, respectively). This supports the hypothesis that the PicOxam^−^ anion undergoes a hydrolysis reaction on the stone surface, as observed when AmPicOxam was reacted with powdered CaCO_3_ (Scheme 2). Accordingly, the conductivity of the solution of compound **2** decreases after stone treatment, while its pH increases as a consequence of the release of 2-picolylamine (Table S6). PXRD experiments were repeated on the surface of the treated samples after 1 year (Figure S22 top). The diffractograms were perfectly reproducible, and the peaks typical of whewellite cannot be spotted, indicating that weddellite had not been converted to whewellite over this period.

The macroscopic properties of the treated samples were analyzed (Table 1) and compared to those of the original samples.

Both surface roughness and colorimetric measurements showed no significant difference between thermally weathered samples and those treated with compound **2**. In particular, the surface roughness shows no significant variations when compared before and after the weathering process (Table S5) and before and after the treatment with compound **2** (Table 1). The variations in the chromatic indices ΔE*_CIE2000_ and ΔE*_CIE1976_ did not significantly exceed the value of 3, indicating that the treatments did not result in color changes that could be perceived by the human eye.

While the samples treated with the 5% *w*/*w* solution showed no changes in the dynamic properties as compared to untreated weathered samples, those treated with the 12% *w*/*w* solution showed a slightly increased cohesion, with an increase in the ultrasonic velocity *v_uts_* and of the dynamic elastic index *E*_d_ (Table 1), partially restoring the properties of the “intact” marble samples (*v_uts_* = 6.2, 1.4, and 2.1 km/s for the “intact”, weathered, and treated samples, respectively).

The hygric behavior of treated and untreated samples was also studied by means of capillarity water absorption and drying tests, in order to determine the effects induced by the treatment with compound **2** on the water-transport properties. The treatment resulted in a slight decrease in the water sorptivity (as testified by the capillary absorption coefficient *CA*) and open porosity of the samples (Table 2 and Figure S23), accompanied by a small but systematic decrease in the apparent density *ρ_a_* due to the lower density of CaOx (2.29 and 2.02 g·cm^−3^ for whewellite and weddellite, respectively) as compared to calcite (2.71 g·cm^−3^) [56].

The cohesion of Carrara marble samples before and after the treatment with compound **2** was evaluated by means pull-off tests. An average maximum pull-off strength of 0.50(1) N·mm^−2^ was determined in the untreated weathered samples. After the treatment, an increase in the strength values up to 0.86(0.2) N·mm^−2^ was determined, indicating an enhanced cohesion and strength of the stone material. Notably, these values testify for a better performance of compound **2** as compared to the results previously reported for AmEtOx [43].

### 2.5. Treatment of Biomicritic Limestone Samples

Biomicrite samples were treated with 5% and 12% *w*/*w* water solutions of compound **2** under the same experimental conditions adopted for the marble samples.

**Figure 3:**
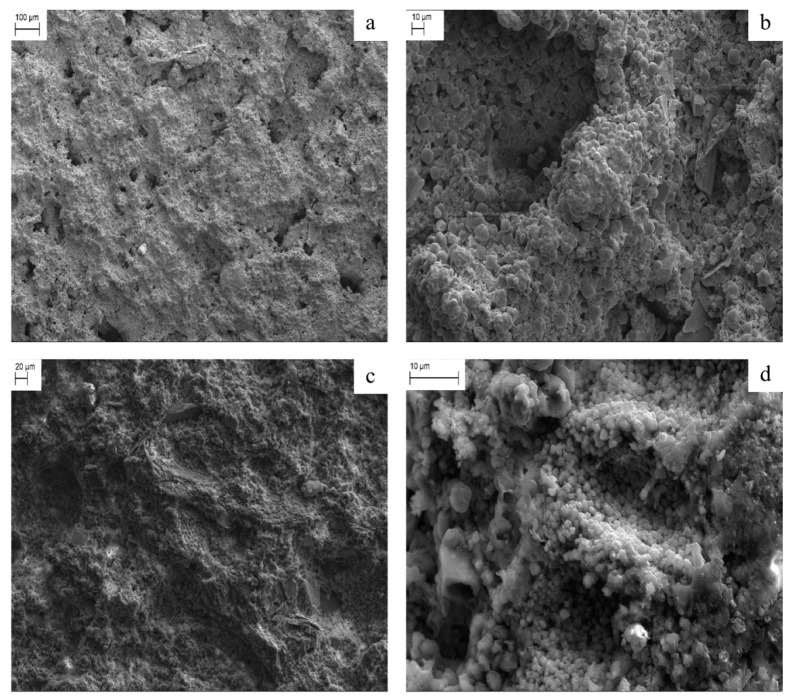
SEM images of biomicrite limestone treated with a 5% (**a**,**b**) and 12% *w*/*w* (**c**,**d**) solution of compound **2**.

The SEM images of the limestone samples treated with a 5% *w*/*w* solution of compound **2** clearly exhibited a deposition of relatively large crystals (~20 μm; Figure 3a,b and Figure S19), similar to those observed on the surface of marble samples treated under the same conditions (see above). In this case, the deposition appeared homogenous due to the absence of micro-cracks. This notwithstanding, an acquisition at higher resolution displays irregularities and defects in the deposited crystals. After treatment with the 12% *w*/*w* solution of compound **2**, the biomicrite surface appeared to be completely covered with a thick homogeneous layer of small crystals (~3–4 μm; Figure 3c, d), without amassing due to the morphology of the sample, with regions where the blending of the consolidant on the spathic cement was clearly detected. A PXRD analysis was carried out both on powdered treated biomicrite samples and directly on the surface a few days after the sample preparation and after one year (Figures S22 bottom, S24 and S25). In all cases, the diffractometric analysis established the deposits as weddellite crystals, in perfect agreement with what was previously reported for the reactions of compound **2** with powdered CaCO_3_ and with marble (Scheme 2). When the PXRD measurements were carried out directly on the stone surfaces, a better characterization of the deposited crystal phases was achieved, thanks to the absence of the amorphous signals due to the grinding effects (Figures S24 and S25). In the samples treated with the 12% *w*/*w* solution, two additional peaks (2*θ* = 7 and 14°, respectively) were detected, possibly due to muscovites or K-feldspars, typical of the composition of biomicrite limestone (Figure S25). As reported above for Carrara marble samples, after the treatments, the conductivity of the water solution decreased, and the pH increased (Table S6).

The macroscopic properties of treated samples are summarized in Table 1. Dynamic properties were not particularly modified by the treatment, suggesting that this should involve only a thin layer without modifying the bulk properties of the sample. Accordingly, more evident was the effect on surface properties, and a variation in the surface roughness was detected upon treatment. The surfaces treated with the 5% *w*/*w* solutions, due to the larger crystalline deposits, show a slight increase in the average roughness measured by the *Rz* parameter. On the contrary, the surface treated with the 12% *w*/*w* solution, due to homogenous coating made of small crystals, was found to be smothered with a remarkable decrease in *Rz* values.

A variation in the colorimetric properties of treated surface with respect to the untreated ones was barely observable, with Δ*E* chromatic variations lower than 5 units (Table 1). In the case of the surface treated with the diluted 5% *w*/*w* solution, an increase in the yellow component *YI* testifies for the opacification discussed above.

Finally, as far as the hygric properties are concerned, treated samples displayed a lower value in the absorption coefficient *CA* as compared to untreated samples (Table 2 and Figure S26) due to a reduced open porosity *P*_o_ and apparent density *ρ*_a_ resulting from the CaOx deposition. Accordingly, the tensile strength remarkably increases, passing from 1.16(2) to 1.56(0.2) N·mm^−2^.

### 2.6. DFT Calculations

Theoretical calculations have proven to be an invaluable tool for understanding the structural, electrochemical, and spectroscopic properties of a variety of compounds, including oxalate and oxamate derivatives [32,33,34,35]. Therefore, the electronic structures of compound **1**, of the PicOxam^−^ anion in compound **2**, and compound **3** were investigated at the density functional theory (DFT) level [57,58]. In order to validate the computational setup, three functionals (B3LYP [59,60,61], mPW1PW [62], and PBE0 [63]) and three basis sets (def2-SVP [64], def2-TZVP [65], and 6-311G [66,67]) were tested in all combinations for compound **1**, and the resulting optimized metric parameters were compared with the structural ones determined by single crystal X-ray diffraction (see above). An evaluation of the sum of root-mean-square deviations calculated for selected distances and angles (RMSD_d_ and RMSD_a_, respectively) showed excellent agreement when the B3LYP/def2-TZVP level of theory was adopted (Tables S7 and S8). This computational setup was thus also used for the optimization of the PicOxam^−^ anion of compound **2** and for compound **3** (Tables S9 and S10), leading to very small RMSD values (RMSD_d_ = 0.522 and 0.244 Å; RMSD_a_ = 0.418 and 0.157° for PicOxam^−^ and compound **3**, respectively). A comparison between optimized and structural bond distances is shown in Figure S27 for compound **1**.

The oxamic portion of all investigated compounds was optimized in a planar conformation, in agreement with what previously was reported for related compounds [34]. The PicOxam^−^ anion was optimized with the pyridine ring rotated by *τ* = 15.9° (Figure S28d; *τ* = 25.1(2)° in the crystal structure of compound **2**). Notably, the very small difference with respect to the completely planar geometry (5.30 kcal·mol^−1^; Figure S28c) testifies a low rotational barrier of the pyridine ring. In the case of compound **3**, the antiperiplanar isomer (Figure S28a), corresponding to the one isolated in the crystal structure, is 8.85 kcal·mol^−1^ more stable than the partially staggered isomer (O–C–C–O dihedral 47.02°; Figure S28b) optimized starting from an antiperiplanar conformation.

**Figure 4:**
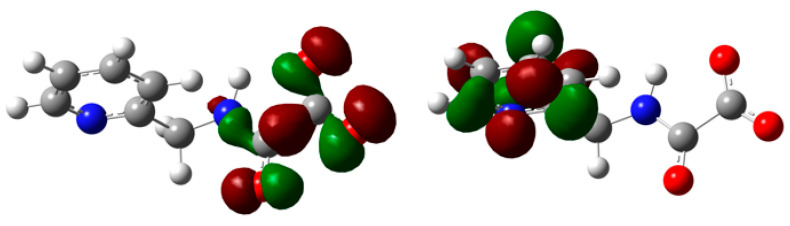
Isosurfaces of KS-HOMO (**left**) and KS-LUMO (**right**) calculated for the PicOxam^−^ anion at B3LYP/def2-TZVP level. Cutoff value = 0.05 |e|.

The Kohn–Sham (KS) HOMO calculated for the PicOxam^−^ anion is largely contributed by the in-plane 2*p* atomic orbitals of the terminal oxygen atoms (Figure 4). The KS-LUMO is a π^*^-MO located on the pyridine ring. The lowest unoccupied MO localized on the oxamic moiety (KS-LUMO + 5) is bonding-in-nature with respect to the C–C bond and closely resembles that previously reported for the Oxam^−^ and PhOxam^−^ anions (Figure S29) [32,35].

The KS-HOMO–5 hosts the lone pair of electrons (LP) of the pyridine ring (Figure S30), which is available for protonation, coordination, or different interactions where the N-donor atom can behave as a Lewis base (*Q*_N_ natural charge [68] on the N-atom: −0.432 |e|). The oxamate carbon C2 (numbering scheme as in Figure S1) shows a remarkably positive charge, suggesting a tendency to undergo hydrolysis reactions increasing on passing from the planar to the rotated conformation (*Q*_C_ = 0.542 |e|, respectively).

A second order perturbation theory analysis of the Fock matrix in an NBO basis [68] showed that the planar form of the oxamate derivatives is stabilized by the delocalization of the LP of electrons on the N-atom of the C(O)NH amide moiety [LP(N) → BD*(C=O) = 75.45, 64.07, and 78.91 kcal·mol^−1^ for compound **1**, the PicOxam^−^ anion, and compound **3**, respectively]. This possibly contributes to the stability of oxamate derivatives in water solution, as proved by the formation of single crystals for X-ray diffraction analysis (see above). On the contrary, previous calculations showed that the interaction with calcite induces a removal of the planarity of the oxamate moiety, thus rendering the oxamate anions more prone to hydrolysis to form the oxalate dianion [45].

## 3. Materials and Methods

Reagents and solvents were used without further purification. The white marble variety “Statuario Michelangelo” from the Apuan Alps and a biomicritic limestone were quarried from Cava Carrarese (Carrara, Italy) and “Cava Flore” in Santa Caterina di Pittinuri (Oristano, Italy), respectively. Prism-shaped specimens of different sizes (2.0 cm × 2.0 cm × 8.0 cm and 2.0 cm × 2.0 cm × 2.0 cm) were sliced. Carrara marble samples underwent a thermal weathering treatment in a Carbolite CWF 1200 muffle furnace, where the temperature was ramped to 500 °C (1 h), held at that temperature for 7 h, and cooled down to 25 °C over a period of 3 h. No artificial weathering treatment was performed on the biomicritic limestone samples.

### 3.1. Physico-Chemical Characterization

Elemental analyses were carried out with a 2400 series II CHNS/O elemental analyzer (T = 925 °C). Melting points were determined using a FALC melting point apparatus mod. C (25–300 °C). FT-IR spectra (KBr beam-splitter, KBr windows, 4000–400 cm^−1^, resolution 4 cm^−1^) were recorded on a Thermo-Nicolet 5700 spectrometer at room temperature on KBr pellets of the samples. Electronic absorption spectra were recorded at 25 °C in a quartz cell (10.00 mm optical path) on a Thermo Evolution 300 (190–600 nm) spectrophotometer. ^1^H-NMR measurements were carried out in CDCl_3_ and DMSO-d*_6_* at 25 °C, using a Bruker Avance III HD 600 MHz (14.1 T) spectrometer at the operating frequency of 600.15 MHz. Chemical shifts are reported in ppm (δ) and are calibrated to the solvent residue. Solubility and *K*_sp_ values were evaluated spectrophotometrically at 25 °C on filtered saturated aqueous solutions, after recording a calibration curve. The pH of the solutions was determined with a Hanna Instruments HI 112 pH meter. Conductivity measurements were carried out with a Crison glp 31 instrument, after calibration with KCl solutions.

### 3.2. Scanning Electron Microscopy

SEM investigations were performed with a Zeiss Evo LS15 microscopy equipped with a LaB_6_ filament as the electron source.

### 3.3. Ultrasonic Measurements

Ultrasonic pulse velocity (*Vp*) measurements were carried out before and after each thermal cycle on a CNS Electronics Pundit tester (accuracy ± 0.1 ms). The 150 MHz (1), 11.82 mm⍉ transducers were attached to the stone surface with Henkel Sichozell Kleister paste (carboxymethyl cellulose) to enhance the transducer–stone coupling. Ten *Vp* measurements were made directly and consecutively at different points along the three orthogonal axes and then averaged. The results were used to calculate the total (*dM*) and relative (*dm*) *Vp* anisotropy indices [69,70,71].

### 3.4. Colorimetric Measurements

Colorimetric measurements were carried out by using a Konica Minolta CM-700d spectrophotometer (illuminant D65), set up to carry out 6 measurements on each point. The results were evaluated as L* (brightness), a* (redness color), and b* (yellowness color) coordinates. The total color difference Δ*E* was calculated according to the CIE-76 and CIE-2000 color space standards [72].

### 3.5. Powder X-ray Diffraction

XRD patterns were collected using coupled θ/2θ scans either on powdered samples or directly on the stone surfaces by means of a Bruker D8 Advance diffractometer equipped with a Cu*K*α X-ray source (40 mW, 40 mA) and a LynxEye XE-T detector (1D high-resolution mode) configured in a Bragg–Brentano geometry. Marble- and limestone-treated samples were analyzed by positioning the bulk samples on a UMC xyz motorized stage. The samples were then correctly aligned with the diffractometer geometry, and data were collected by keeping a fixed sample illumination length of 18 mm by means of motorized slits. PXRD measurements were carried out after the treatment of the stone sample with AmPicOxam aqueous solution and after 1 year to verify the eventual evolution of the coating.

### 3.6. Single Crystal X-ray Diffraction

Data collection and processing for compounds **1**–**3** were performed using CrysAlisPro (Rigaku Oxford Diffraction) [73]. The crystals were suspended in perfluoroether oil, then a crystal was selected and mounted on a MiTeGen loop. Single-crystal X-ray diffraction data of compound **1** were collected on a Rigaku FRE+ diffractometer, equipped with VHF Varimax confocal mirrors and an AFC12 goniometer and HyPix 6000 detector. Data for compound **2** were collected on Rigaku 007HF diffractometer, equipped with Varimax confocal mirrors, an AFC11 goniometer, a HyPix 6000 detector, and an Oxford Cryosystems low temperature device. Diffraction data for compound **3** were collected on a Rigaku FRE+ diffractometer, equipped with HF Varimax confocal mirrors and an AFC12 goniometer and HG Saturn 724+ detector. The crystals were kept at a steady *T* = 100(2) K during data collection. The structure of compound **3** was already published and deposited at CSD with code POWGEQ. These structures were then solved with the ShelXT [74] structure solution program (using dual space algorithm) and by using Olex2 [75] as the graphical interface, with their models refined with version 2018/3 of ShelXL [76], using least squares minimization on *F*^2^.

### 3.7. Hygric Measurements

Real density (*ρ_r_*), bulk density (*ρ_a_*), compactness index (*I_c_*), and the open porosity index (*ϕ%*) were determined by a water saturation experiment carried out on specimens brought to constant mass in an oven at *T* = 50 ± 2 °C. Weighed samples were placed in a container full of distilled H_2_O for 24 h. After this lapse of time, the specimens were weighed on the hydrostatic balance and again on the analytical balance after having gently buffered the surfaces. Based on the three masses measured, it was possible to calculate these structural parameters by means of the following relationships:

$$\rho_{r}=\frac{m_{i}}{\left(m_{i}-m_{idr}\right)}\;\;\;\;\;\left[\frac{g}{{cm}^{3}}\right]\;\;\;\;\;\rho_{a}=\left(\frac{m_{i}}{\left(m_{i}-m_{hydr}\right)}\;\right){\times \rho }_{H_{2}O}\;\;\;\;\left[\frac{g}{{cm}^{3}}\right]$$

$$I_{c}=\left(\frac{\rho_{r}}{\rho_{a}}\right)\times 100\;\;\;\;\;\;\;\;\;\;\phi \%=\left[\frac{\left(m_{ps}-m_{i}\right)}{\left(m_{ps}-m_{hydr}\right)}\right]\times 100$$

where *m_i_* is the initial mass of the dry specimen, *m_ps_* the mass after saturation with water, and *m_hydr_* the mass weighed on the hydrostatic balance. The real density (*ρ_r_*) was also determined using a Micromeritics AccuPyc II 1340 Helium pycnometer, equipped with a 3.5 cm^3^ sample holder cell. The measurements were carried out on fragments of Carrara white marble and biomicritic limestone of 1 cm^3^ < *V* < 2 cm^3^. For each type of treatment, three fragments were selected, on which five density measurements were performed.

Capillary water uptake tests were performed in accordance with the European Standard UNI EN 15801:2010 [77]. The samples were dried at 60 °C for 24 h. The water absorption curve is expressed as *Q* (kg·m^−2^) for the *Y*-axis vs. the square root of the absorption time (*h_1_*_/*2*_) for the *X*-axis. The slope of the curve in the initial steep region is the capillary absorption coefficient *CA*, calculated as:

$$CA=\frac{Q_{i}-Q_{0}}{t_{1}^{-\frac{1}{2}}}$$

Drying experiments were carried out according to NorMaL 29/88 [78]. Soaked samples were weighed at increasing time intervals. The drying index (*DI*) was calculated as follows:

$$DI=\frac{\int_{t_{i}}^{t_{f}}f\left(wt\right)dt}{{wt}_{max}\cdot t_{f}}$$

Over a time-interval ranging between *t_i_* and *t_f_*, *wt_max_* is the maximum water content at initial testing time [79]. Vacuum water absorption tests were performed on all samples according to UNI EN 1936:2010 (pressure = 0.2 kPa) [80]. From this free-water saturation method through Archimedes’ principle and buoyancy techniques, the open porosity (*P_o_*) of the stone samples can also be determined by [6]:

$$P_{o}=\frac{m_{s}\;-\;m_{d}}{m_{s}-m_{h}}\cdot 100$$

where *m_d_*, *m_h_*, and *m*_s_ are the mass values of the dry and saturated specimen in water and air, respectively. From these measurements, the real (*ρ_r_*) and apparent density (*ρ_a_*) were determined [81].

### 3.8. Physico-Mechanical Measurements

Pull-off tests were performed according to standard UNI EN 1015-12:2000 and used to evaluate the resistance to tearing of the sample [82]. Steel stubs, 20 mm in diameter, were grit blasted and attached to samples (marble cubes of 2.0 cm × 2.0 cm × 2.0 cm and biomicrite cubes of 3.0 cm × 3.0 cm × 3.0 cm) with an epoxy adhesive to form a butt joint. After the curing of the adhesive, the joints were then pulled in a universal testing machine fitted with a 5 kN load cell and tested at a rate of 2 mm/min at 25 °C. Strength values were then determined. The surface roughness of the specimens was investigated by means of a Mitutoyo SJ-201 portable surface roughness tester. Two faces for each specimen were analyzed and for each ten measurements (1 mm slipped from each other) were carried out.

### 3.9. QM Calculations

Theoretical calculations were carried out at the density functional theory (DFT) level [57,58] with the Gaussian 16 commercial suite of programs [83]. The choice of functional and basis set was carried out by adopting all combinations of the B3LYP [59,60,61], mPW1PW [62], and PBE0 [63] functionals along with the def2-SVP [65], def2-TZVP [65], and 6-311G [66,67] basis set. The B3LYP hybrid functional was eventually adopted along with def2-TZVP basis set. Harmonic frequency calculations were carried out to verify the nature of the energy minima at the optimized geometries by verifying the absence of significative negative frequencies. Natural charge distributions [68] were calculated at the optimized geometries at the same level of theory. The programs GaussView 6.0.16 [84] and Molden 7.3 [85] were used to investigate the optimized structures and molecular orbital shapes.

### 3.10. Synthesis

#### 3.10.1. Synthesis of *O*-methyl-*N*-(pyridin-2-ylmethyl)oxamate (**1**)

To a dimethyloxalate (5.00 g, 0.042 mol) methanol solution (20 mL), a 2-picolylamine solution (4.3 mL, 0.042 mol) in the same solvent (10 mL) was added dropwise. The solution was refluxed for 24 h and then concentrated at reduced pressure. The resulting oil was purified by column chromatography (stationary phase: silica gel; eluent acetonitrile/ethanol 9:1) and compound **1** was obtained as a white solid. M.p. 83 °C. Yield 3.64 g, 69.5%. UV-Vis-NIR (CH_3_CN): λ_max_ (ε) = 223 (5500), 249 (4100), 254 (4300), 260 (4100), 267 (2800 M^−1^·cm^−1^) nm. FT-IR (KBr pellet): ῦ = 3172 (m), 3013 (w), 2958 (vw), 1739 (s), 1700 (s), 1596 (m), 1525 (m), 1480 (w), 1430 (m), 1356 (w), 1307 (m), 1235 (s), 1214 (m), 1174 (w), 1146 (vw), 1099 (w), 1045 (w), 1009 (m), 992 (m), 940 (w), 840 (w), 771 (s), 621 (m), 509 (w) cm^−1^. ^1^H-NMR (CDCl_3_) δ = 8.57 (d, 1H, Ar); 8.27 (s, 1H, NH); 7.68 (td, 1H, Ar); 7.27 (d, 1H, Ar); 7.24 (dt, 1H, Ar); 4.65 (d, 2H, CH_2_); 3.92 (s, 3H, CH_3_) ppm. Elemental analysis (calcd. for C_9_H_10_N_2_O_3_): C, 55.53 (55.67); H, 5.01 (5.19); N, 14.38 (14.43)%.

#### 3.10.2. Synthesis of Ammonium *N*-(pyridin-2-ylmethyl)oxamate (**2**)

To a water solution (70 mL) of compound **1** (12.8 g, 0.066 mol), a water solution of ammonium hydrogen carbonate (8.00 g, 0.096 mol) was slowly added, and the resulting mixture was refluxed for 4 h. The hot solution was poured in a glass plate, and the solvent was air evaporated. The solid was washed with dichloromethane. Yield: 11.34 g (87.7%). M.p. 195 °C. UV-Vis (H_2_O): λ_max_ (ɛ) = 260 (4180 M^−1^·cm^−1^) nm. FT-IR (KBr pellet): ῦ = 3300 (s), 3051 (w), 2408 (w), 2158 (w), 1674 (s), 1620 (s), 1528 (m), 1475 (w), 1418 (s), 1263 (m), 1233 (m), 1021 (w), 1002 (w), 848 (w), 753 (w), 618 (w), 808 (w), 487 (w), 455 (w) cm^−1^. ^1^H-NMR (DMSO-d_6_) δ = 8.63 (t, 1H, NH), 8.49 (d, 1H, Ar), 7.73 (m, 1H, Ar), 7.43 (br s, 4H, NH_4_^+^), 7.25 (d, 2H, Ar), 4.35 (d, 2H, CH_2_) ppm. Elemental analysis (calcd. for C_8_H_11_N_3_O_3_): C 48.67 (48.73), H 5.63 (5.62), N 21.09 (21.31)%.

#### 3.10.3. Synthesis of *N*,*N*′-bis(pyridin-2-ylmethyl)oxalamide (**3**)

Compound **3** was synthesized according to a variation of the method described by Liu and co-workers [86]. To a dimethyloxalate aqueous solution (2.04 g, 0.017 mol in 10 mL of distilled water), an excess of 2-picolylamine (6.8 mL, 0.068 mol in 7 mL of distilled water) was added dropwise. The resulting solution was refluxed over a period of 3 h. The product was filtered, washed with distilled water and petroleum ether, and dried at 40 °C. Yield 2.02 g (45%). M.p. 159 °C. UV-Vis-NIR (CH_3_CN): λ_max_ (ε) = 202 (6130), 259 (2170 M^−1^·cm^−1^) nm. FT-IR (KBr pellet): ῦ = 3289 (s), 3049 (vw), 3012 (vw), 2933 (vw), 1652 (vs), 1591 (m), 1570 (w), 1528 (s), 1471 (m), 1433 (m), 1243 (m), 1219 (m), 994 (w), 757 (s), 638 (m), 616 (m), 519 (m) cm^−1^. ^1^H-NMR (CDCl_3_): δ = 8.57 (d, 2H, Ar), 8.41 (br s, 2H, NH), 7.66 (td, 2H, Ar), 7.27 (d, 2H, Ar), 7.21 (dt, 2H, Ar), 4.64 (d, 4H, CH_2_) ppm. Elemental analysis (calcd. for C_14_H_14_N_4_O_2_): C 62.15 (62.21), H 4.76 (5.22), N 20.73 (20.73)%.

### 3.11. Reaction of Compound ***2*** with CaCO_3_ Powder

A weighed amount (5.91 g, 0.0299 mol) of compound **2** dissolved in 150 mL of bi-distilled water (4% *w*/*w*) was added to 1.50 g (0.0150 mol) of anhydrous CaCO_3_. The suspension was stirred for 24 h at room temperature. The solid was collected by filtration. FT-IR (KBr pellet): ῦ = 3470 (m), 2980 (vw), 2868 (vw), 2511 (w), 1796 (m), 1649 (s), 1414 (s), 874 (s), 769 (w), 700 (s), 613 (vw), 508 (vw), 465 (vw), 403 (w) cm^−1^.

### 3.12. Coating Preparation

Thermally treated stone samples were immersed in 50 mL of freshly prepared 5% *w*/*w* or 12% *w*/*w* aqueous solutions of compound **2** into a static batch for 24 h [41,42,43,44,45,46]. The solutions (100.00 g) were prepared by dissolving a weighed amount of compound **2** into distilled water (C = 0.253 M and 0.608 M for the 5% *w*/*w* or 12% *w*/*w* solutions, respectively). The pH of the solution was measured (pH = 6.5), and the samples were washed with water, dried at room temperature for 4 days, and then kept into a thermostatic heater (60 °C) for 24 h.

## 4. Conclusions

In this paper we reported on the synthesis, characterization, and treatment tests of the novel salt ammonium *N*-(pyridin-2-ylmethyl)oxamate (AmPicOxam, **2**) as a conservation agent for Carrara marble and biomicritic limestone. Compound **2** readily reacts with calcite to give calcium oxalate dihydrate (weddellite) and thus represents an unconventional, economically affordable, and more soluble precursor of calcium oxalate than the commonly used ammonium oxalate. Worthy of note, the complete hydrolysis of AmPicOxam occurs during the application step, and no traces of the original salt were found on the treated stone samples. This prevents subsequent reactions of the treated stone surface with ions in the groundwater and the release of persistent contaminants that could have potentially negative environmental effects. The coating features are strongly dependent on the microstructure of the used stone, particularly on the open porosity, specific surface, and crystallo-clastic grain size. AmPicOxam overcomes two limiting features of AmOx: (i) being remarkably more soluble in water (with a concentration of saturated aqueous solution, 1.00 M, more than double than that of ammonium oxalate, 0.4 M) and (ii) providing solutions with pH values close to neutrality and therefore not aggressive towards the treated surfaces. Remarkable variations in the uniformity of the coating depending on the adopted concentration of compound **2** were observed, the most uniform and homogeneous coating being achieved with the 5% *w*/*w* solution. The treatment on both Carrara marble and biomicritic samples resulted in an improvement of their cohesion (as indicated by pull-off tests), increasing the intrinsic strength of the material against further degradation processes. In addition, the inertness of calcium oxalate towards acidic agents represents a remarkable protection for artifacts exposed outdoors. Notably, the variations of open porosity and apparent density determined on treated specimens with respect to the untreated weathered marble and biomicrite limestone samples indicate AmPicOxam as a more efficient precursor of CaOx, not only with respect to AmOx but also to previously investigated CaOx precursors, such as AmEtOx [43]. The consolidation effect was supplemented by no appreciable alterations in color or vapor permeability, thus testifying the great compatibility of the treatment with the stone substrates. PXRD analyses carried out on the same marble and biomicrite samples one year after the treatments confirm the composition of the surface deposition layer, testifying for its stability over time. DFT calculations gave an insight into the electronic properties of the PicOxam^−^ anion, responsible on the one hand for the stability of compound **2** in water solution and on the other for the tendency to hydrolysis when interacting with carbonate stone substrates.

The outcome of this research establishes the salts of monoamide derivatives of oxalic acid as a very promising class of new materials for the conservation of carbonate stone materials. A comparison with the results reported previously on AmOxam and AmPhOxam demonstrates the subtle role played by the substituent in tailoring the chemical–physical properties and reactivity of the resulting ammonium salts.

## Figures and Tables

**Table 1:** Petrographic, dynamic, structural, and colorimetric macroscopic properties of stone samples before and after treatment with compound **2**.

		Weathered White Carrara Marble	Treated Marble (5% *w*/*w*)	Treated Marble (12% *w*/*w*)	Biomicrite Limestone	Treated Biomicrite (5% *w*/*w*)	Treated Biomicrite (12% *w*/*w*)
Petrography	Mineralogic composition	Calcite, muscovite	Calcite, muscovite, weddellite	Calcite, muscovite, weddellite	Calcite, quartz, muscovite, microcline, albite, chamosite	Calcite, quartz, muscovite, microcline, albite, chamosite, weddellite	Calcite, quartz, muscovite, microcline, albite, hamosite, weddellite
Dynamics	*t_uts_* (µm)	52.4 ± 0.1	56.0 ± 0.1	38.9 ± 0.1	24.0 ± 0.1	27.6 ± 0.1	26.0 ± 0.1
	*v_uts_* (km/s)	1.5 ± 0.1	1.4 ± 0.1	2.1 ± 0.2	3.0 ± 0.2	2.9 ± 0.2	2.7 ± 0.2
	*E_d_* (MN/m^2^)	4.23 ± 0.08	3.55 ± 0.06	7.57 ± 0.19	20.3 ± 0.8	18.8 ± 0.7	16.6 ± 0.6
Structure	*ρ_a_* (g/cm^3^)	2.62 (0.01)	2.63 (0.01)	2.63 (0.01)	1.70 (0.04)	1.72 (0.02)	1.75 (0.03)
	*ρ_r_* (g/cm^3^)	2.708 (0.003)	2.705 (0.006)	2.711 (0.003)	2.71 (0.01)	2.689 (0.001)	2.686 (0.003)
	I_c_ %	96.75	97.23	96.83	62.73	63.96	65.15
	*ɸ %*	3.0 (0.4)	2.8 (0.2)	3.1 (0.5)	29 (2)	30.7 (0.5)	29 (1)
Colorimetry	L*	92.90	92.60	92.40	80.30	79.20	75.60
	a*	0.25	0.26	−0.15	2.40	3.10	2.16
	b*	0.26	3.70	4.91	17.70	21.70	17.14
	*C*	3.08	3.71	4.91	17.86	21.92	17.28
	*WI_CIELAB76_*	68.56	64.92	7.38	–	–	–
	*YI_ASTME313_*	6.15	53.92	9.52	37.55	45.57	38.00
	ΔE*_CIE1976_	–	0.70	0.56	–	4.21	2.29
	ΔE*_CIE2000_	–	3.13	2.27	–	4.74	3.35
Roughness	*Ra* (µm)	2.6 (0.3)	2.8 (0.2)	2.8 (0.4)	15 (1)	17 (2)	14 (3)
	*Rz* (µm)	27 (5)	30 (5)	19 (4)	122 (20)	137 (8)	76 (11)
	*Rq* (µm)	3.4 (0.4)	3.8 (0.3)	4 (1)	19 (2)	21 (3)	17 (3)

**Table 2:** Capillary absorption coefficient *CA* (kg·m^−2^·h^−1/2^), drying index *DI* (% h^−1^), open porosity P_o_ (%), relative *ρ_r_*, and apparent density *ρ_a_* (g·cm^−3^) determined by water transport tests for thermally weathered Carrara marble and biomicritic limestone before and after treatment with compound **2**. Standard deviations are reported in parentheses.

	*CA*	Δ*CA*	*DI*	Δ*DI*	*P*_o_	Δ*P*_o_	*ρ*_r_	Δ*ρ*_r_	*ρ*_app_	Δ*ρ*_app_
Carrara white marble										
Untreated	7.16	–	31.86	–	5.70(4)		2.6(0.2)	–	2.7(0.2)	
Treated with **2**	4.76	−33.5%	27.61	13.33%	4.0(4)	–30%	2.9(0.1)	1.5%	3.2(0.2)	–19%
Biomicritic limestone										
Untreated	13.22	–	3.95	–	41.0(3)	–	2.8(0.2)	–	1.7(0.1)	–
Treated with **2**	9.01	−31.8%	2.76	−30.1%	38.0(3)	–7%	2.3(0.1)	–18%	1.3(0.1)	–24%

## Data Availability

Crystallographic data were deposited at CCDC (CIF deposition number 2269589 and 2269588 for compounds **1** and **2**·1/2H_2_O, respectively).

## Supplementary Materials

The following supporting information can be downloaded at: https://www.mdpi.com/article/10.3390/molecules28155768/s1, Figures S1–S3: molecular drawings from XRD experiments; Figures S4–S9, S12–S14: spectroscopic measurements (compounds 1–3); Figure S10: spectroscopic determination of the solubility of compound 2; Figures S11, S15, S20, S21, S22, S24 and S25: powder X-ray diffractograms; Figure S16: thermal treatment diagram for Carrara marble; Figures S17 and S18: optical microscopy images; Figure S19: SEM images; Figures S23 and S26: water capillary absorption/desorption curves; Figure S26–S30: DFT optimized geometries and selected KS-MOs. Tables S1–S4: crystallographic data and structural results; Table S5: stone characterization; Table S6: pH and conductivity solution measurements; Table S7–S10: DFT-optimized geometries.

## Sample Availability

Samples of the compounds **1**–**3** are available from the authors upon request.
