# Supplementary material for: Ammonium N-(pyridin-2-ylmethyl)oxamate (AmPicOxam): A Novel Precursor of Calcium Oxalate Coating for Carbonate Stone Substrates
Source: Molecules. 2023 Jul 30;28(15):5768. doi: 10.3390/molecules28155768 (PMC10421195; doi:10.3390/molecules28155768)
Supplement: Supplementary file 1 [file molecules-28-05768-s001.zip › molecules-2477877-supplementary.pdf]

Supplementary Materials

Ammonium *N*-(pyridin-2-ylmethyl)oxamate (AmPicOxam): a novel precursor of calcium oxalate coating for carbonate stone substrates

Anna Pintus,[1] M. Carla Aragoni,[1] Gianfranco Carcangiu,[2] Veronica Caria,[1] Simon J. Coles,[3] Eleanor Dodd,[3] Laura Giacopetti,[1] Domingo Gimeno,[4] Vito Lippolis,[1] Paola Meloni,[5,6] Simone Murgia,[1] Antonia Navarro Ezquerra,[7] Enrico Podda,[1,8] Claudia Urru,[1] and Massimiliano Arca[1,*]

[1] Università degli Studi di Cagliari, Dipartimento di Scienze Chimiche e Geologiche, S. S. 554 bivio per Sestu, 09042 Monserrato (Cagliari), Italy.
[2] Consiglio Nazionale Delle Ricerche (CNR), Istituto di Scienze dell'Atmosfera e Del Clima (ISAC), UOS di Cagliari c/o Dipartimento di Fisica, Università degli Studi di Cagliari, S. S. 554 bivio per Sestu, 09042 Monserrato (Cagliari), Italy
[3] National Crystallography Service, School of Chemistry, University of Southampton, Southampton SO17 1BJ, UK
[4] Facultat de Ciències de la Terra, Universitat de Barcelona, c/ Martí i Franquès s/n, 08028 Barcelona, Spain
[5] Dipartimento di Ingegneria Meccanica, Chimica e dei Materiali, via Marengo 2, 09123, Cagliari, Italy
[6] Laboratorio Colle di Bonaria, Università degli Studi di Cagliari, Via Ravenna snc, Cagliari, 09125, Italy
[7] Departamento de Tecnología de la Arquitectura, EPSEB-UPC, Avda. Doctor Marañón, 44-50, 08028 Barcelona, Spain
[8] Centro Servizi di Ateneo per la Ricerca (CeSAR), Università degli Studi di Cagliari, S.S. 554 bivio Sestu, 09042 Monserrato (Cagliari), Italy

***** Correspondence: marca@unica.it

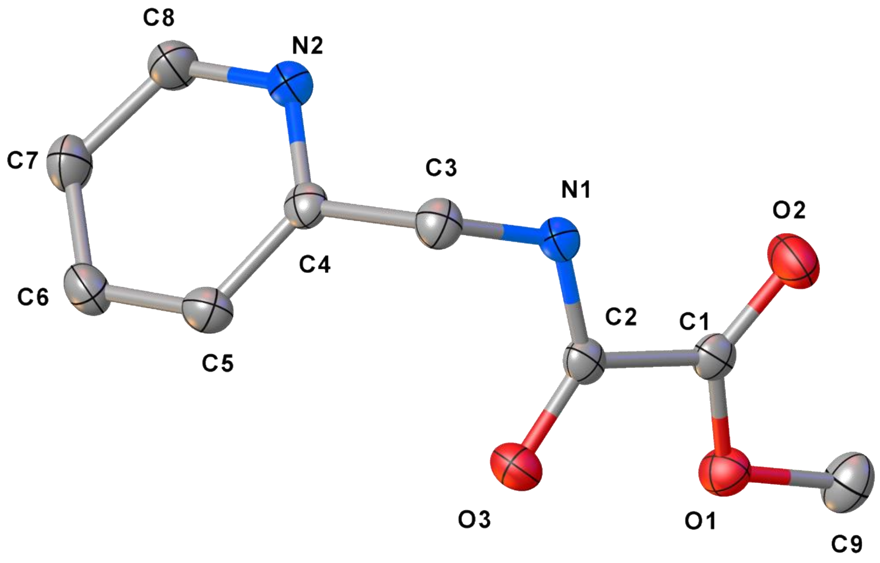

Figure S1. Molecular structure and atom labelling scheme of compound **1**. Thermal ellipsoids were drawn at 50% probability level. Hydrogen atoms were omitted for clarity.

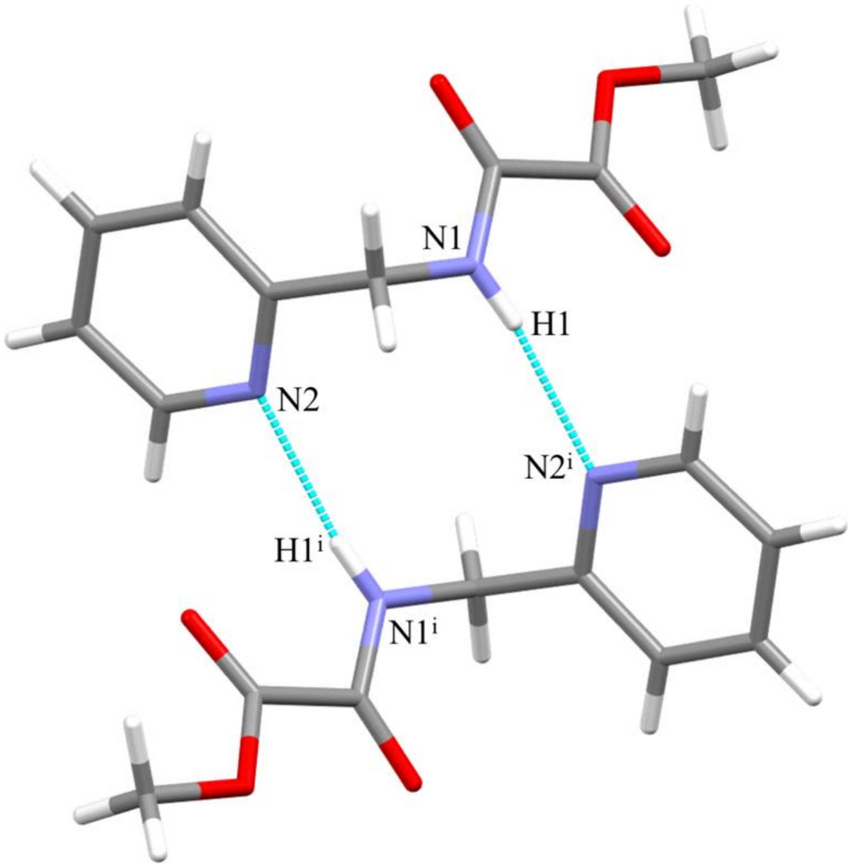

Figure S2. H-bonding interactions between pairs of *O*-methyl-*N*-(pyridine-2-ylmethyl)oxamate units in compound **1**. ^{i} = 1–x, 1–y, 1–z.

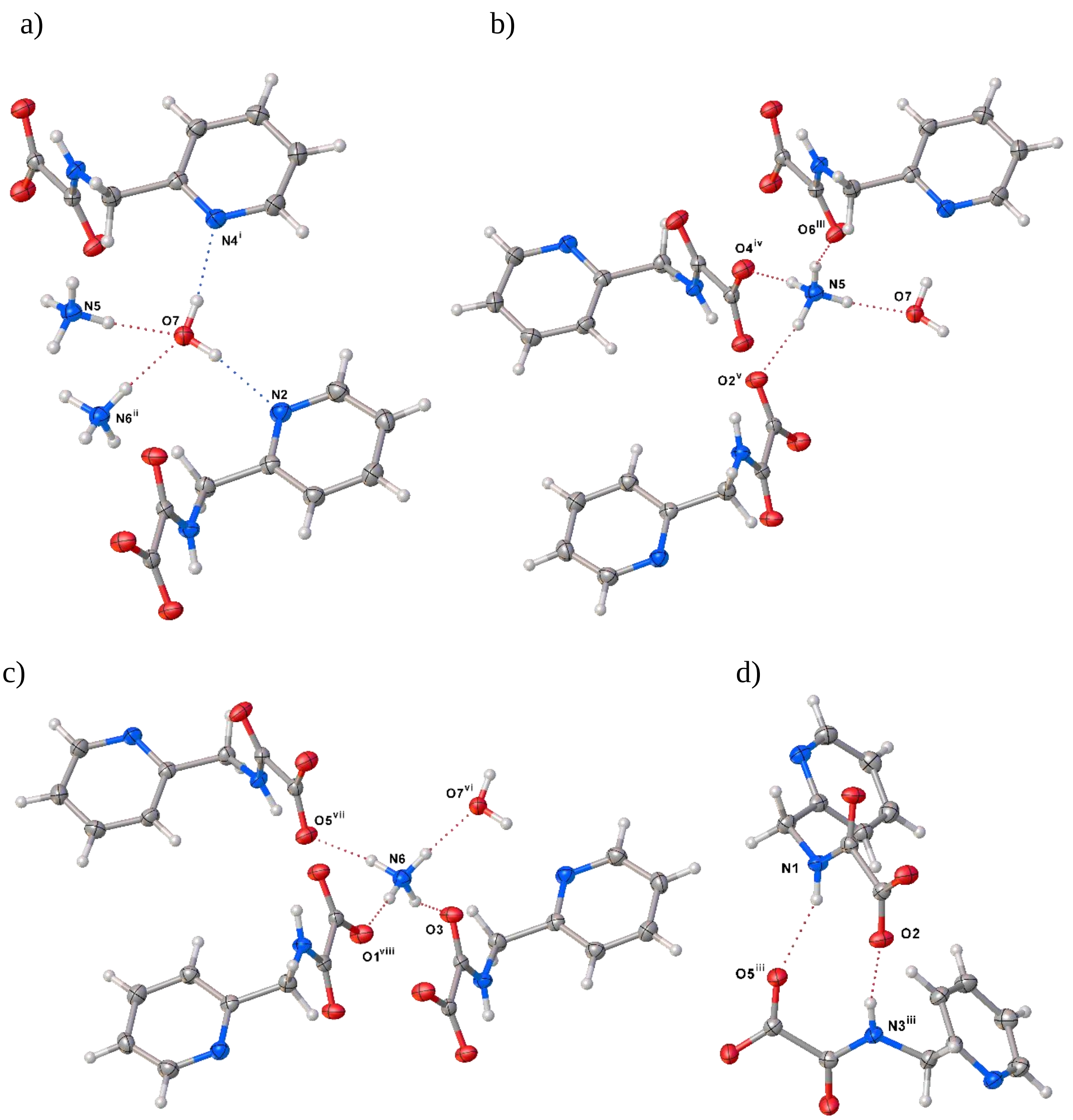

Figure S3. H-bonding interactions in compound **2**·1/2H_2O involving the co-crystallized water molecule (a), the two independent ammonium ions (b, c), the two independent interacting *N*-(pyrin-2-ylmethyl)oxamate anions (d). Thermal ellipsoids were drawn at 50% probability level. Symmetry codes: ^{i} = −1−x, 1−y, −z, ^{iii} = −x, 1−y, −z; ^{iv} = −1+x, 1+y, +z; ^{v} = −x, 2−y, 1−z; ^{vi} = 1+x, +y, +z; ^{vii} = +x, 1+y, +z; ^{viii} = 1−x, 2−y, 1−z.

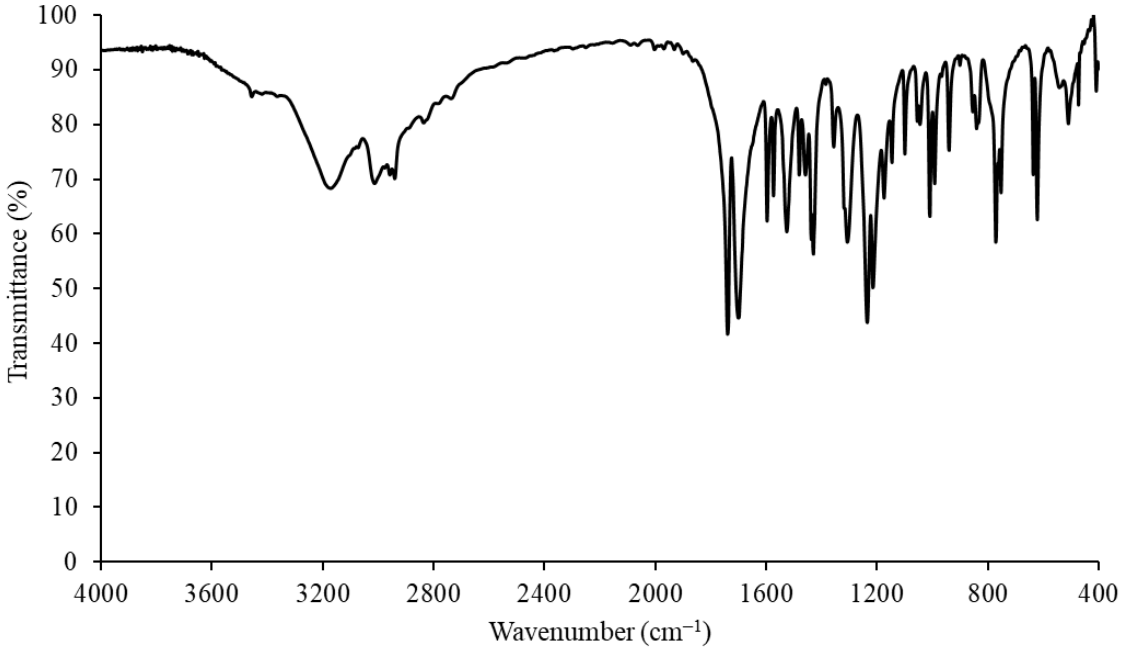

Figure S4. Solid state FT-IR spectrum (4000–400 cm^{-1}) recorded for compound **1** in KBr pellet.

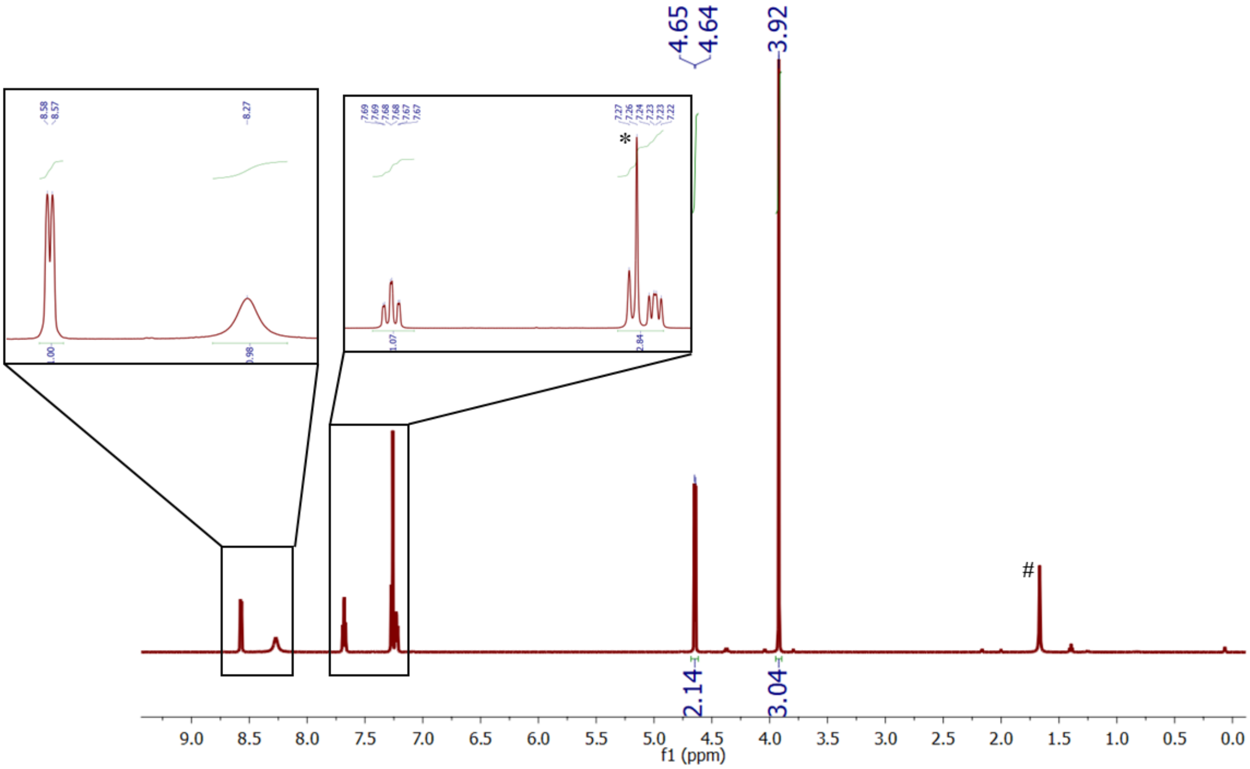

Figure S5. ^{1}H-NMR spectrum recorded for compound **1** in $CDCl_3$ solution. Marked peaks correspond to the solvent residual signal (*) and water (#).

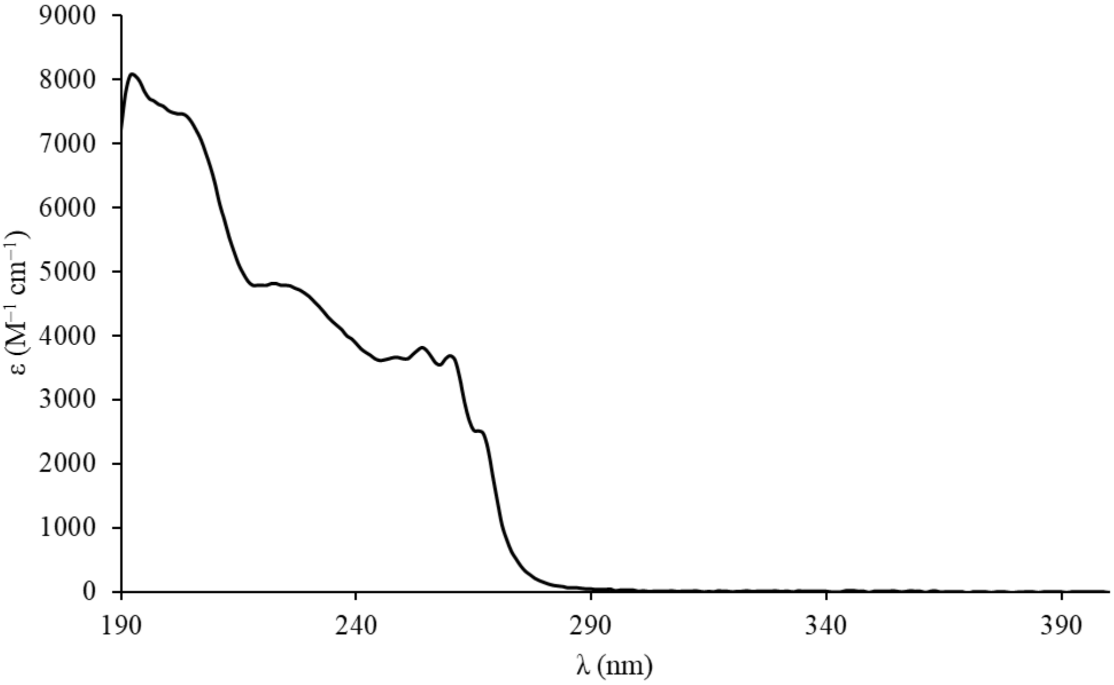

Figure S6. UV-Vis spectrum (190–400 nm) recorded for compound **1** in acetonitrile solution.

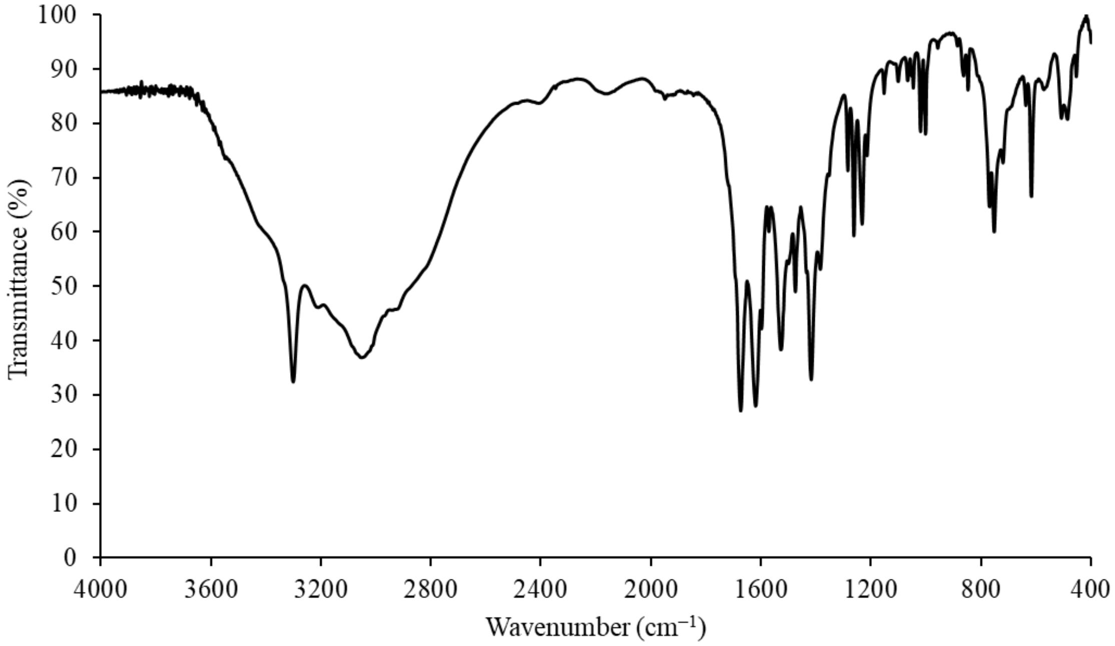

Figure S7. Solid state FT-IR spectrum (4000–400 cm^{-1}) recorded for compound **2** in KBr pellet.

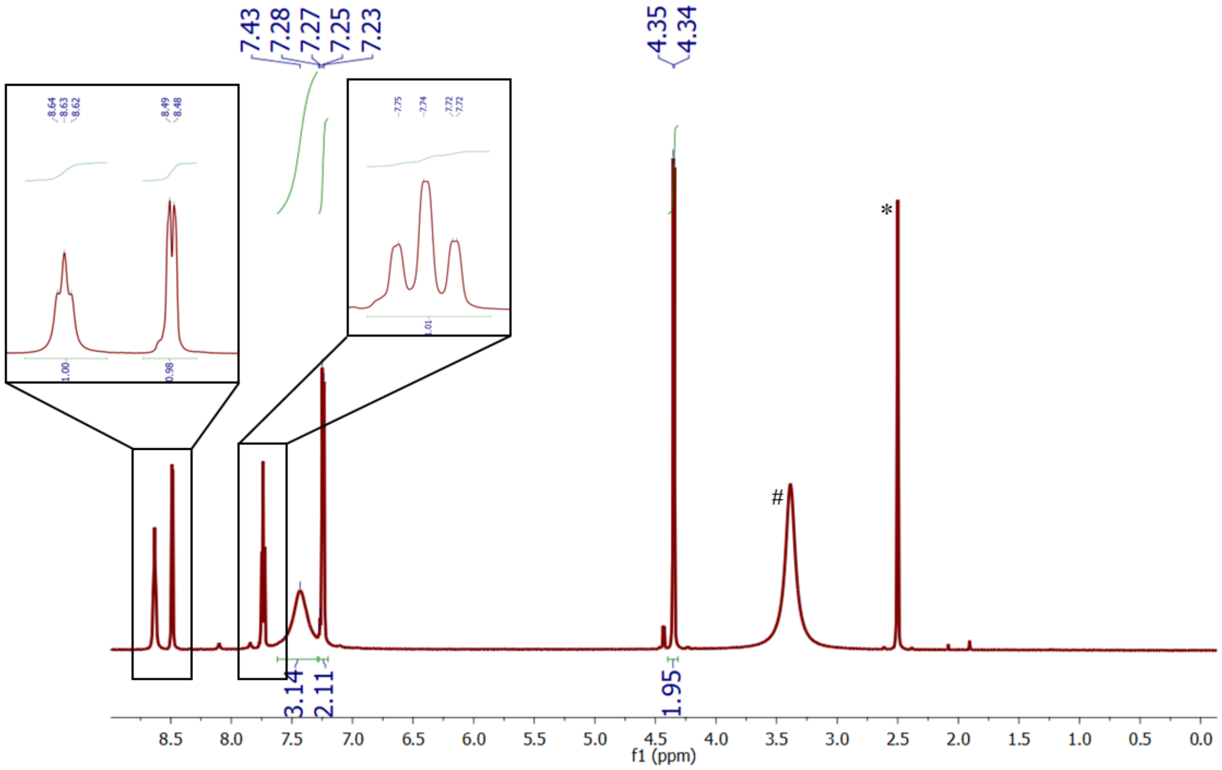

Figure S8. ^{1}H-NMR spectrum recorded for compound **2** in DMSO-d_6 solution. Marked peaks correspond to the solvent residual signal (*) and water (#).

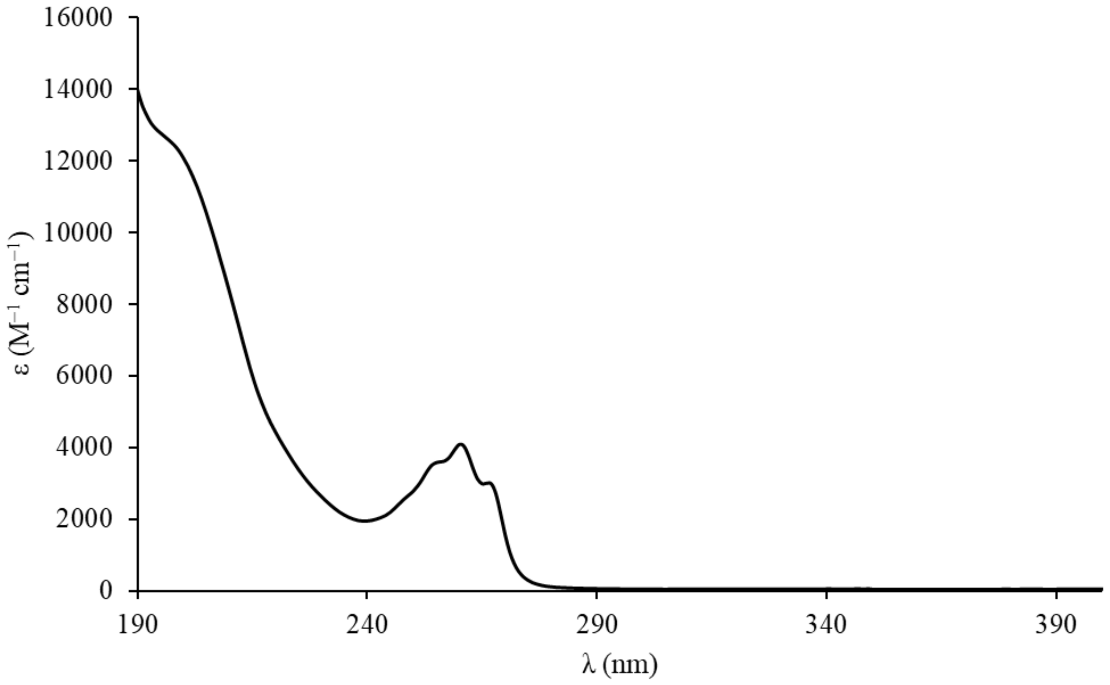

Figure S9. UV-Vis spectrum (190–400 nm) recorded for compound **2** in water solution.

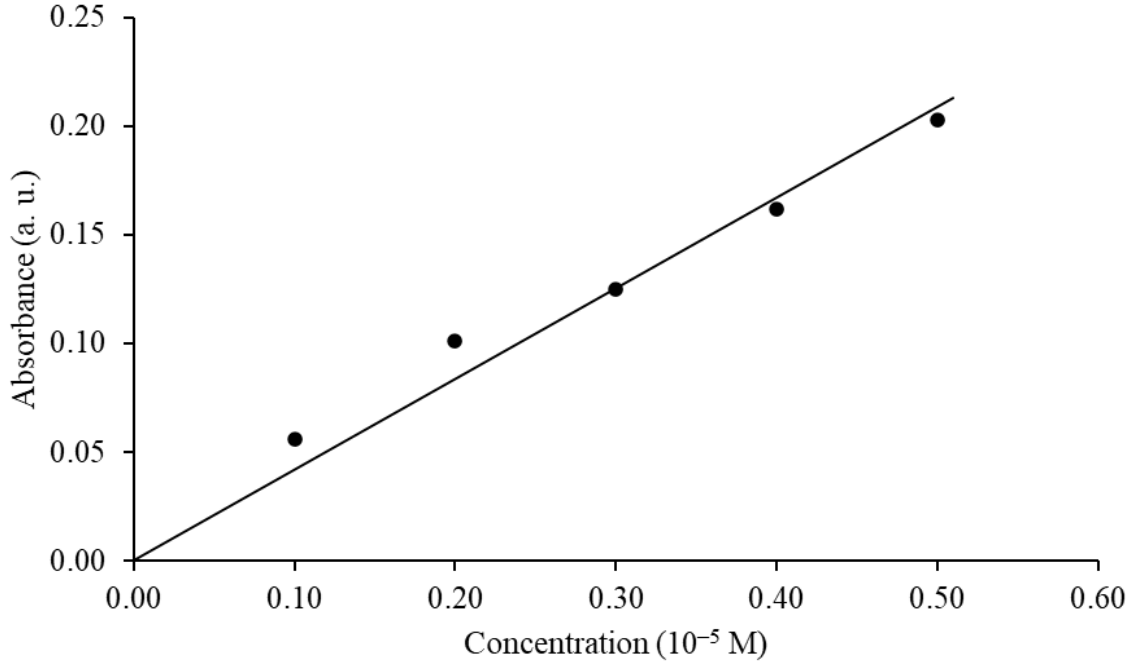

Figure S10. Absorbance (λ = 260 nm) recorded for compound **2** as a function of molar concentration in water solution. The resulting molar extinction coefficient ε (4170 $M^{-1}\cdot cm^{-1}$) was used for evaluating the solubility from the molar concentration of a filtered saturated solution (correlation coefficient R = 0.997).

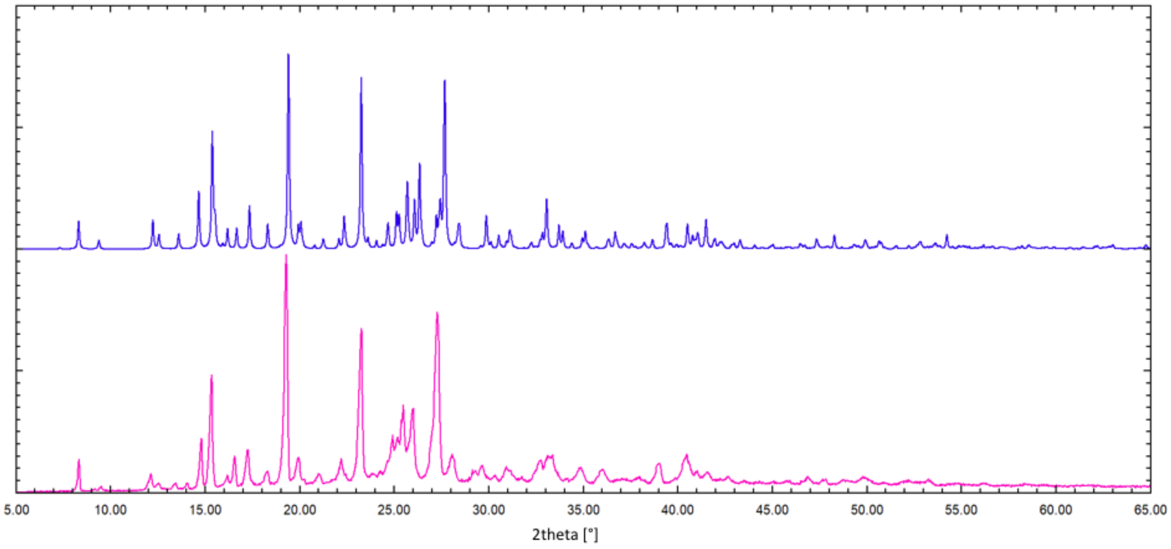

Figure S11. Comparison between experimental XRD pattern of compound **2** (magenta) and the calculated pattern from single crystal diffraction analysis (blue).

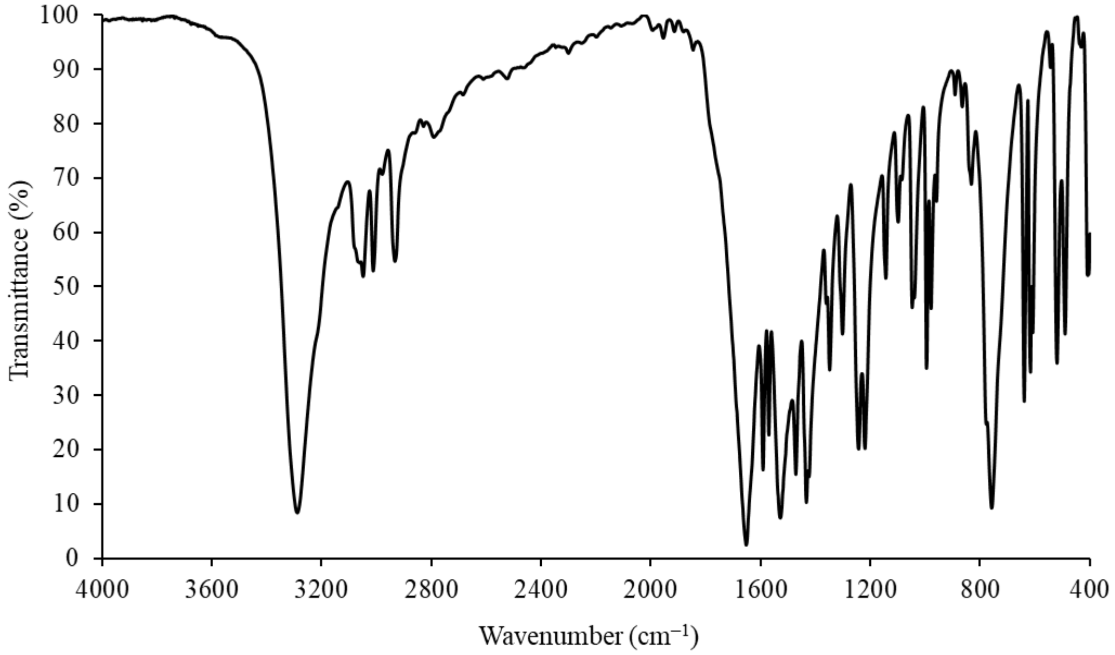

Figure S12. Solid state FT-IR spectrum (4000–400 cm^{-1}) recorded for compound **3** in KBr pellet.

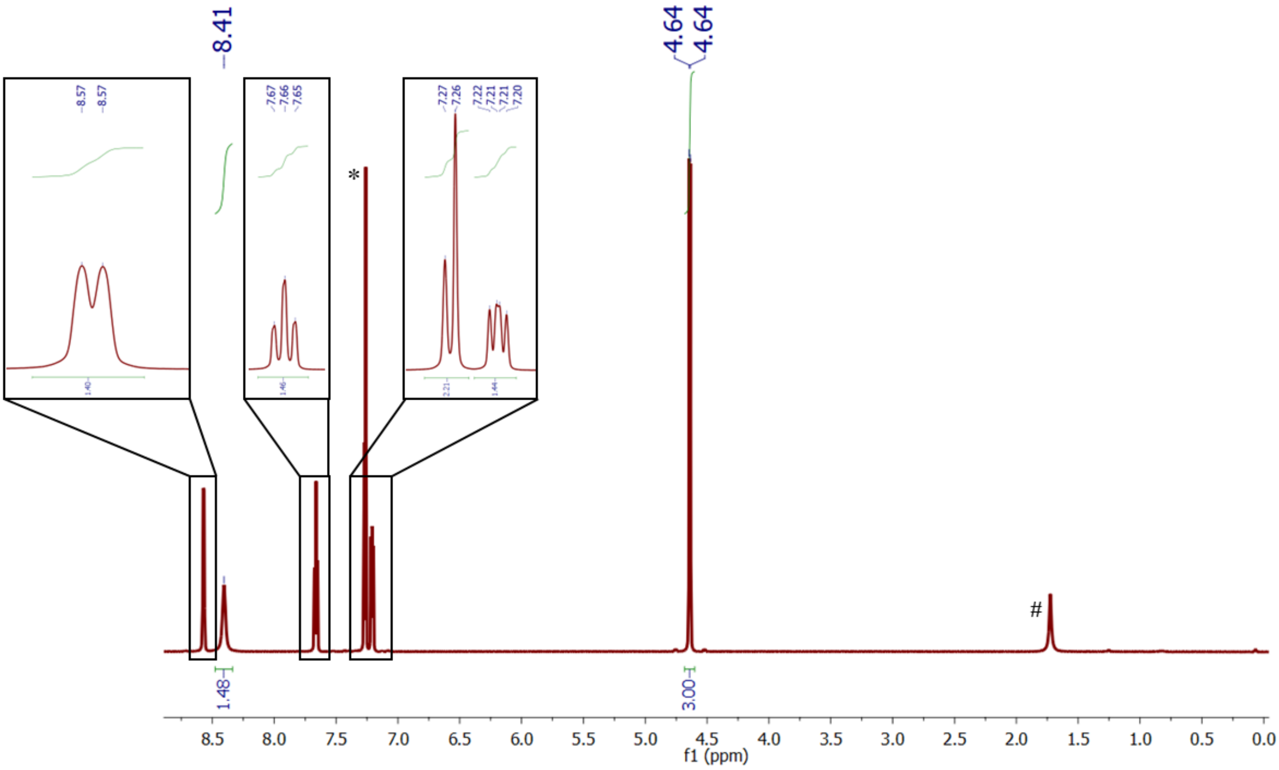

Figure S13. ^{1}H-NMR spectrum recorded for compound **3** in $CDCl_3$ solution. Marked peaks correspond to the solvent residual signal (*) and water (#).

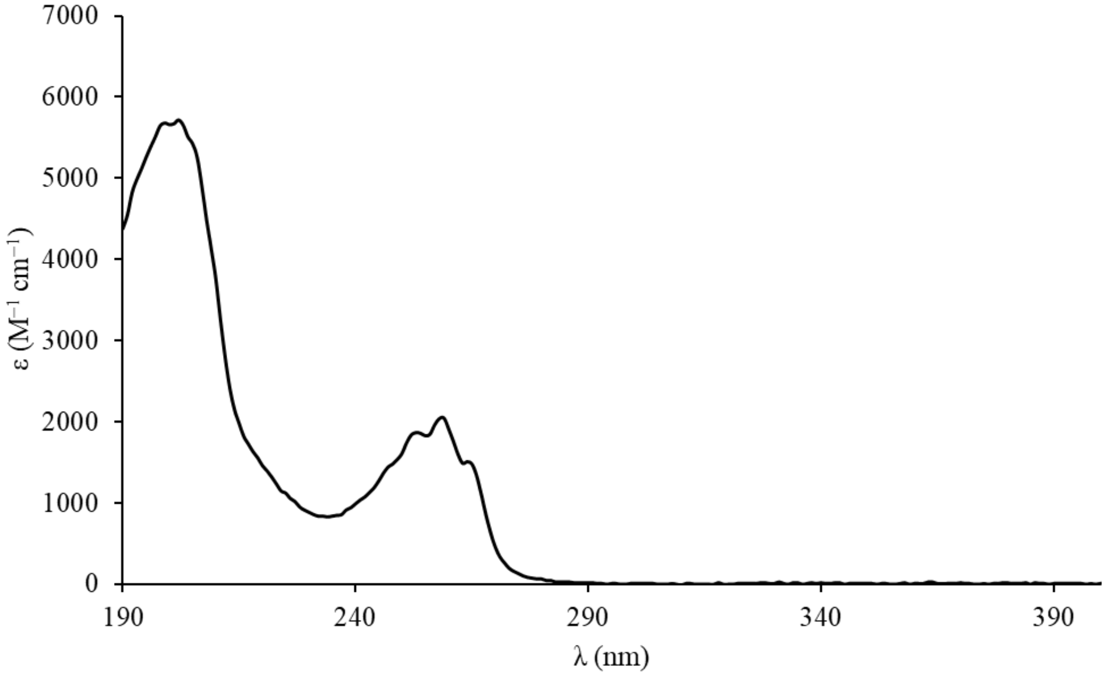

Figure S14. UV-Vis spectrum (190–400 nm) recorded for compound **3** in acetonitrile solution.

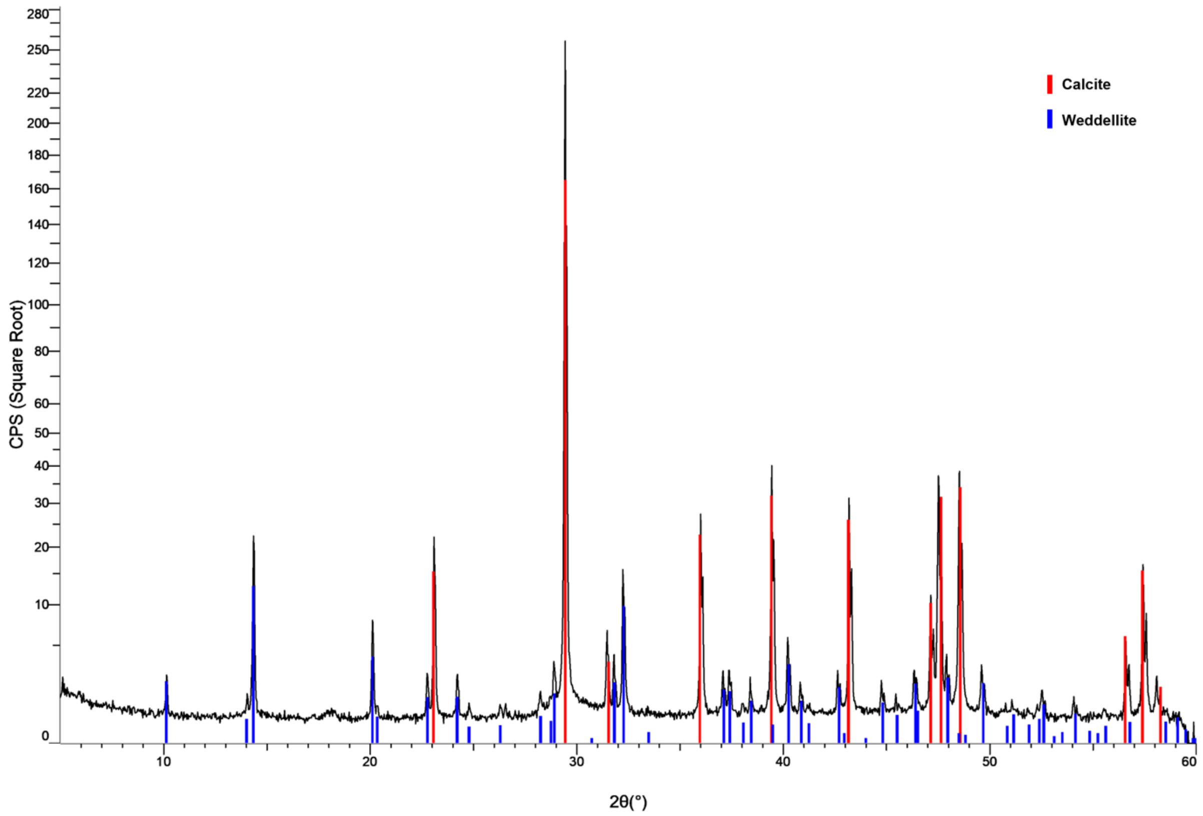

Figure S15. Powder X-ray diffractogram recorded on the product of the reaction performed between calcium carbonate and compound **2** in 1:2 molar ratio for 24 h.

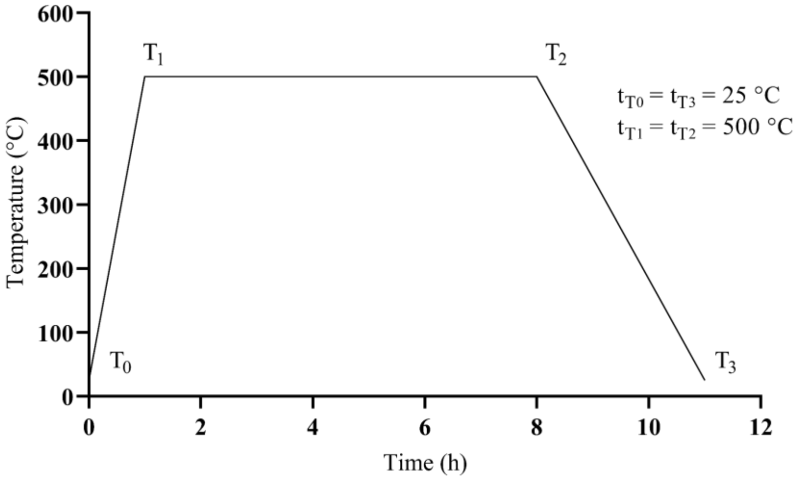

Figure S16. Thermal treatment diagram for the artificial weathering of Carrara marble.

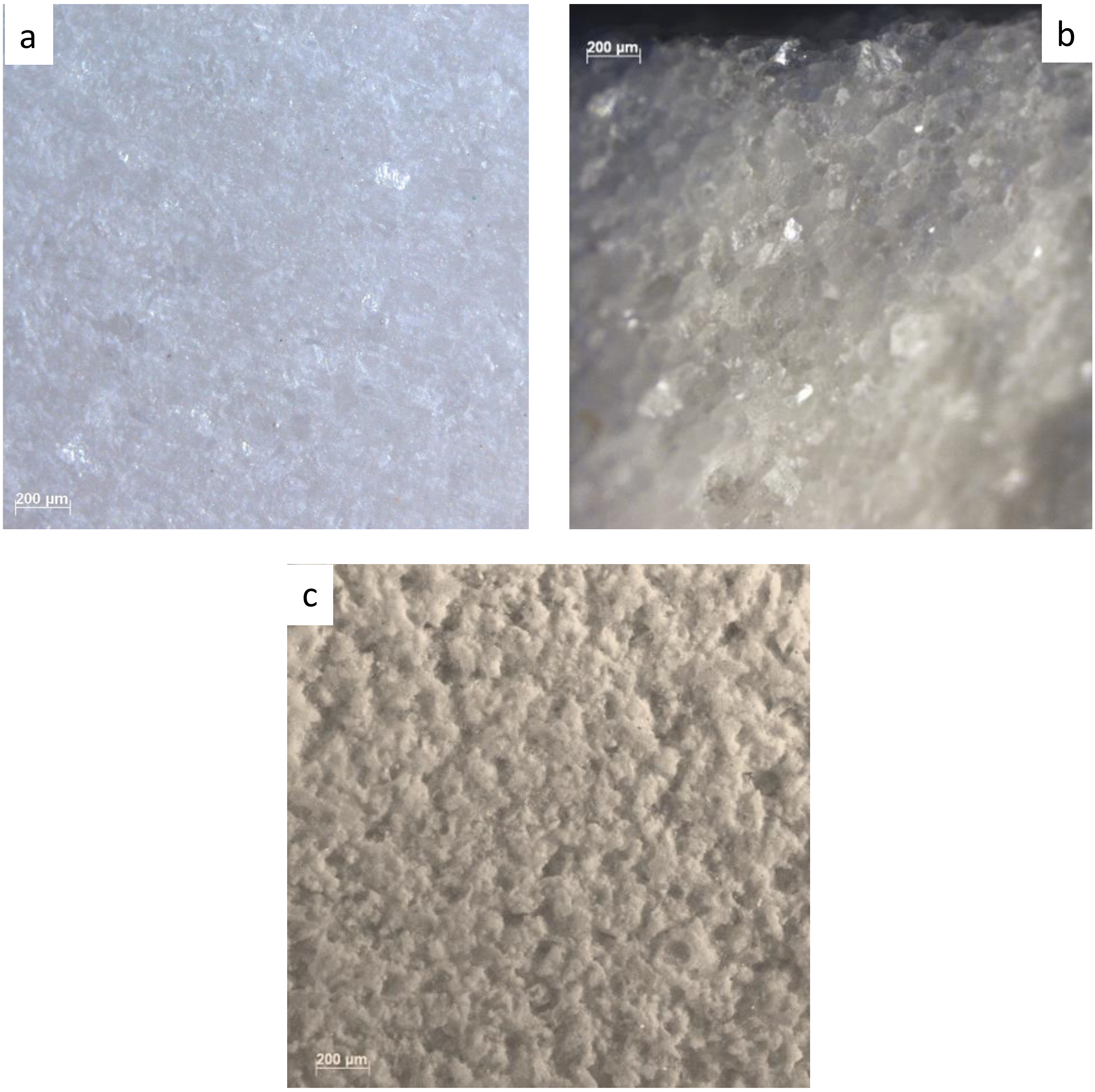

Figure S17. Optical microscopy images of Carrara marble (top) before (a) and after (b) the weathering process and biomicrite limestone (bottom, c).

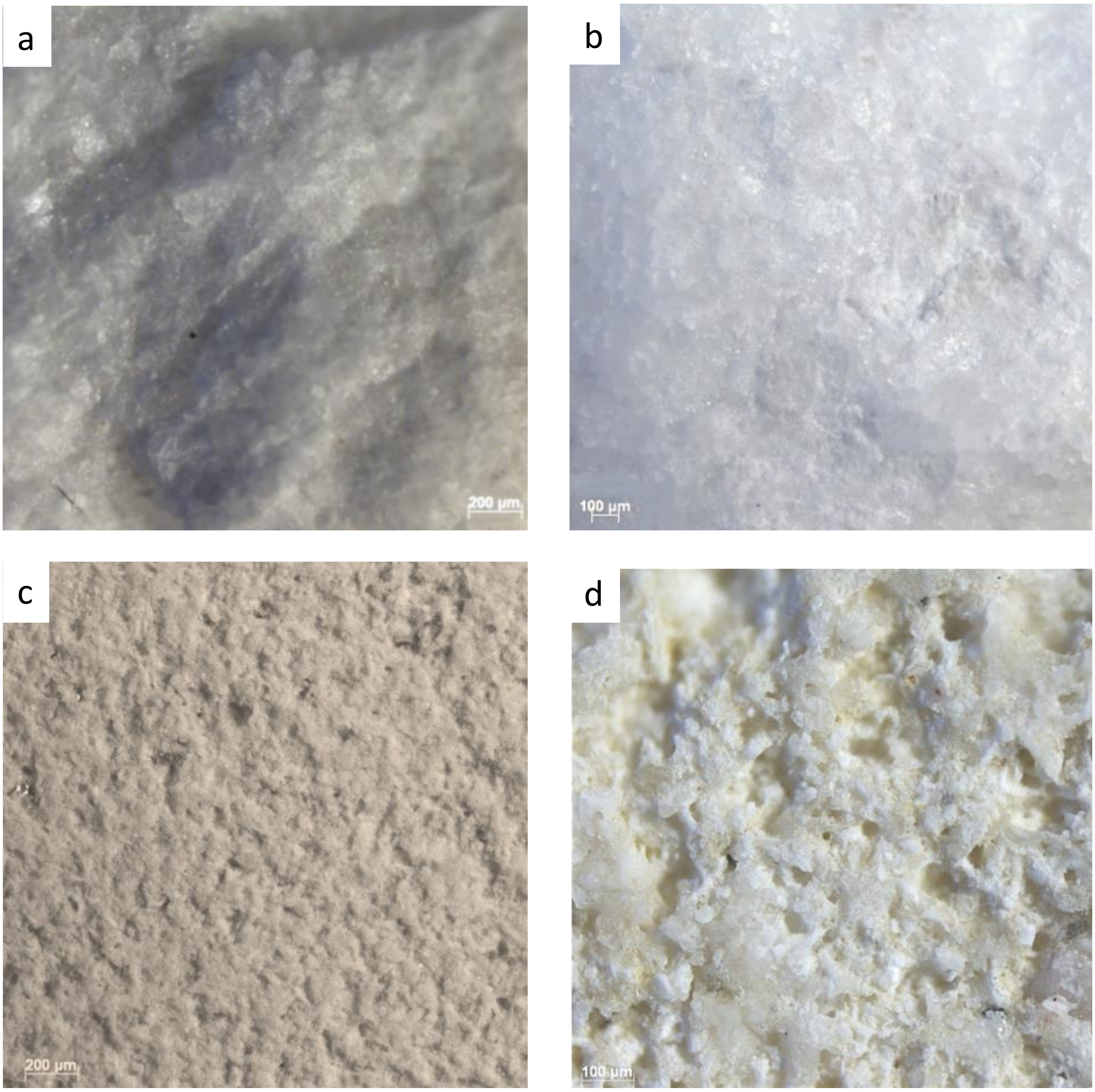

Figure S18. Optical microscopy images of Carrara marble (a, b) and biomicritic limestone (c, d) treated with a 5% w/w (a, c) and 12% w/w (b, d) water solution of compound **2** in static batch.

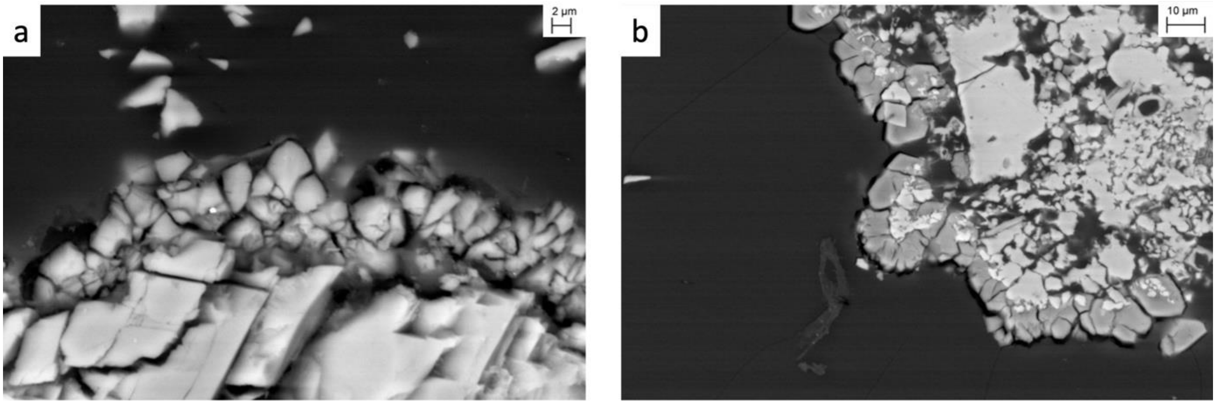

Figure S19. Detail overview of the surface coating on treated marble (a) and biomicrite limestone (b) treated with a 5% w/w water solution of compound **2**.

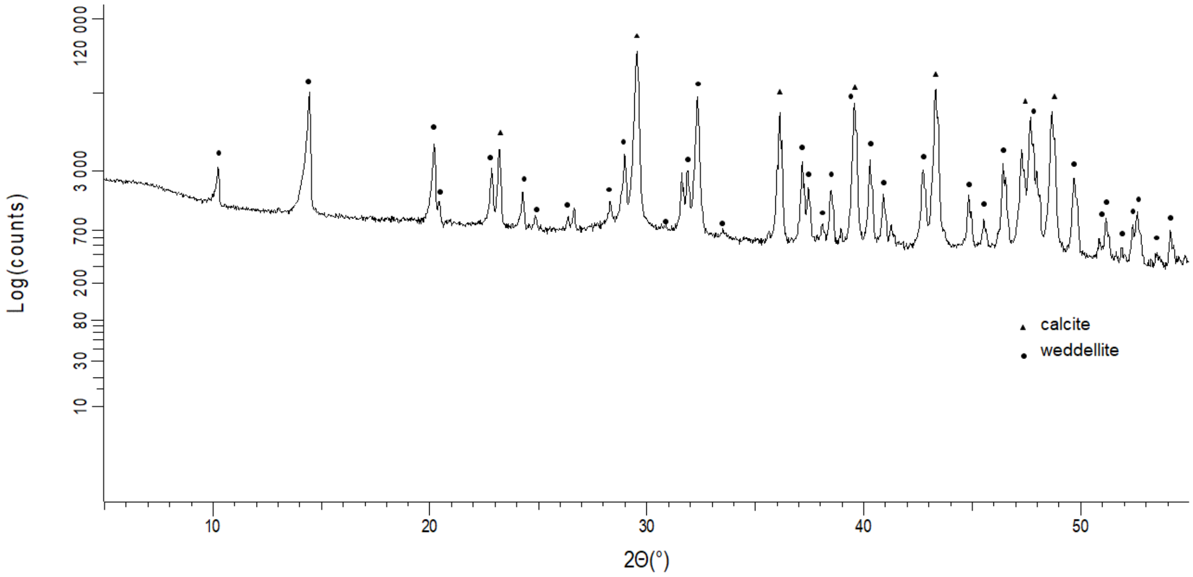

Figure S20. XRD diffractogram of a powdered sample of Carrara marble treated with a 5% w/w solution of compound **2**.

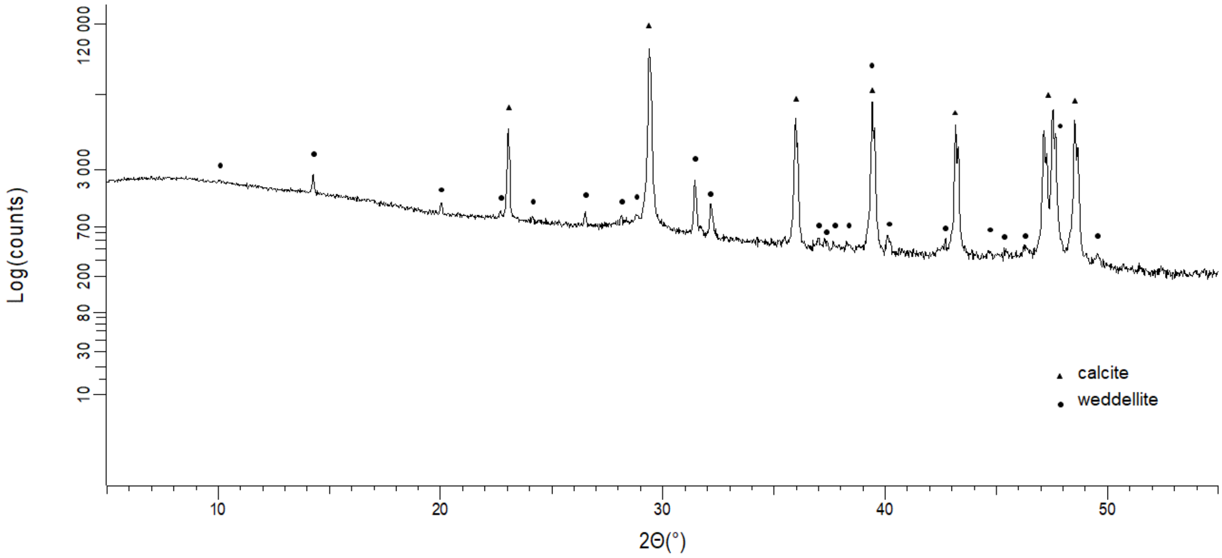

Figure S21. XRD diffractogram of a powdered sample of Carrara marble treated with a 12% w/w solution of compound **2**.

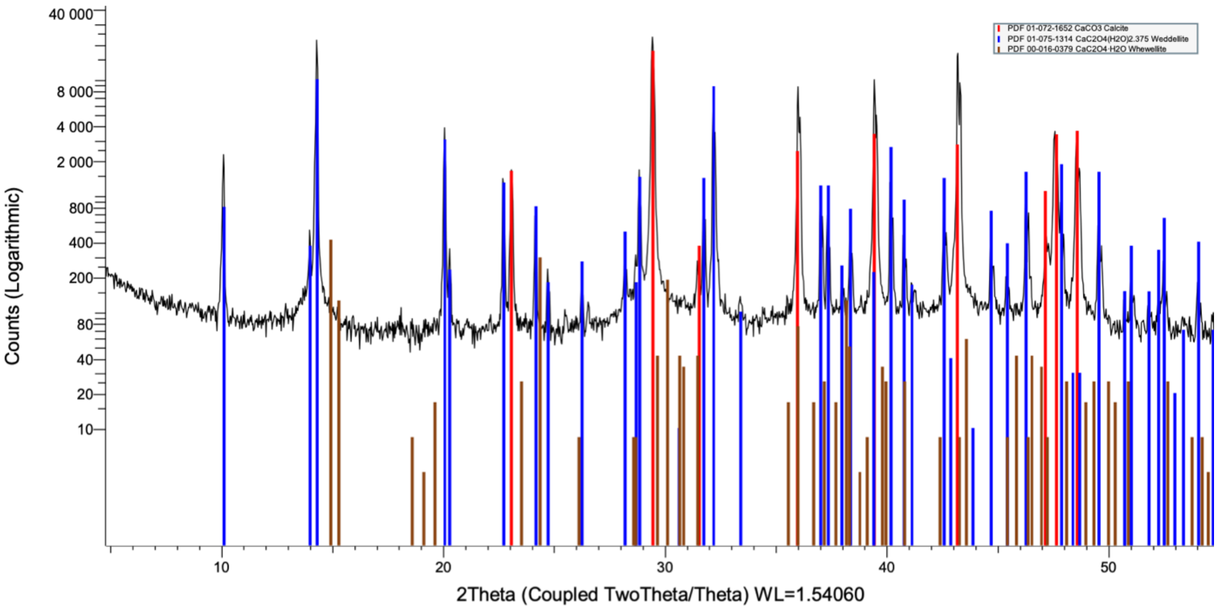

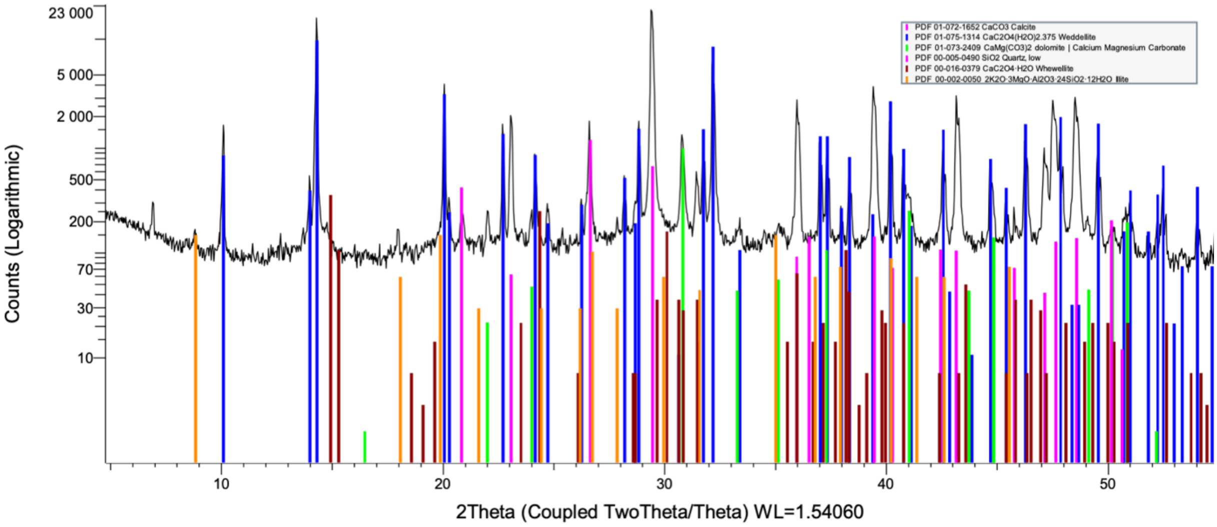

Figure S22. XRD diffractogram on the surface of Carrara marble (top) and biomicrite limestone (bottom) samples one year after the treatment with a 12% w/w solution of compound **2**. In both diffractograms whewellite (brown) cannot be spotted.

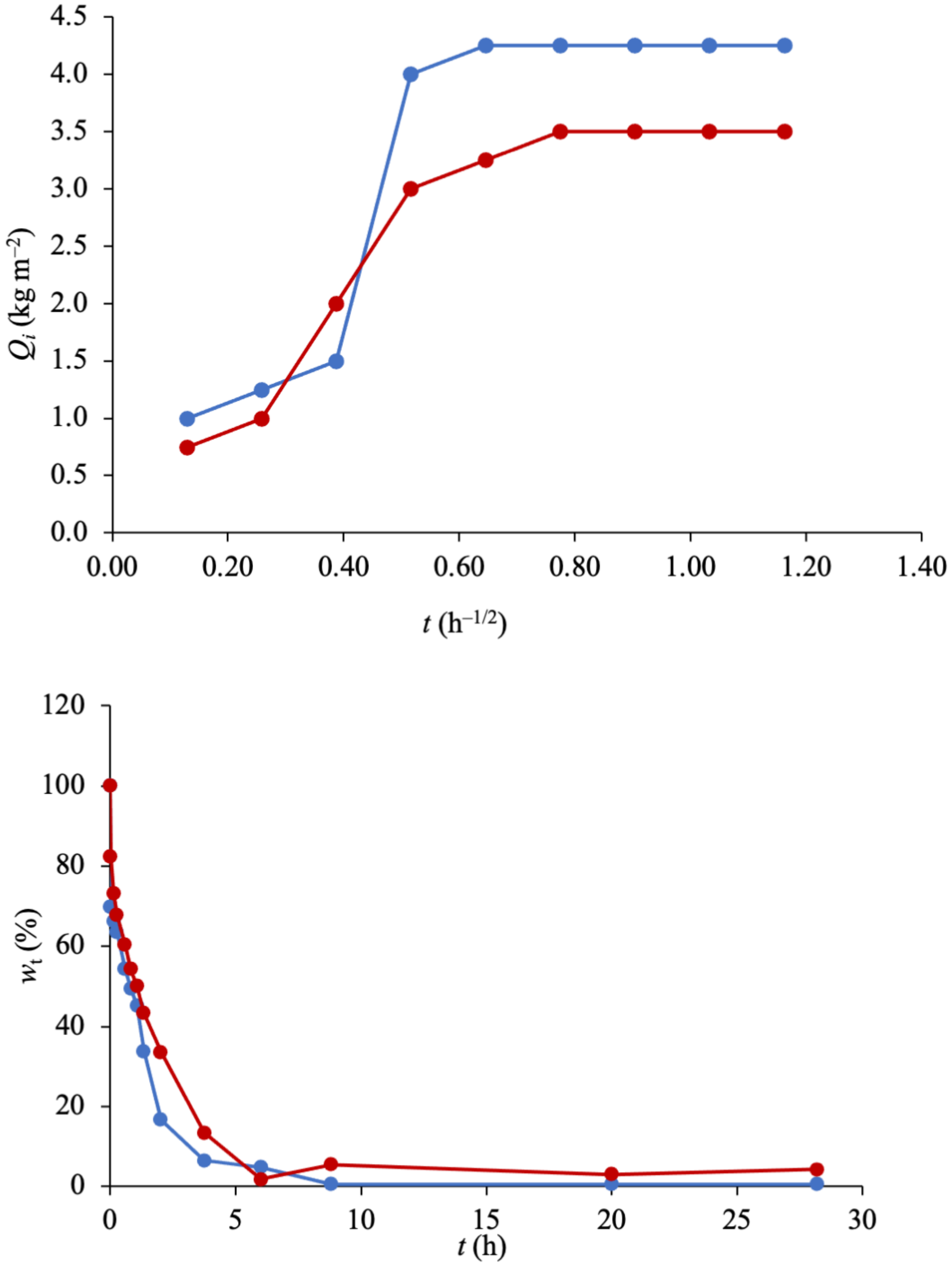

Figure S23. Water capillary absorption (top) and desorption (bottom) curves for thermally weathered Carrara marble samples before (blue) and after (red) the treatment with compound **2** solution.

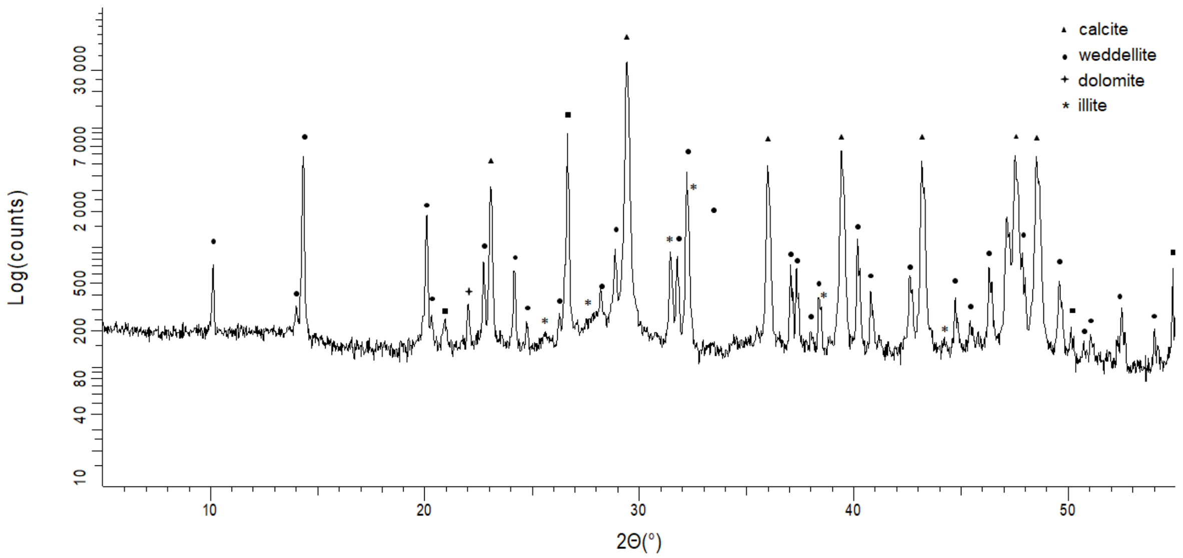

Figure S24. XRD diffractogram of a powdered sample of biomicrite limestone treated with a 5% w/w solution of compound **2**.

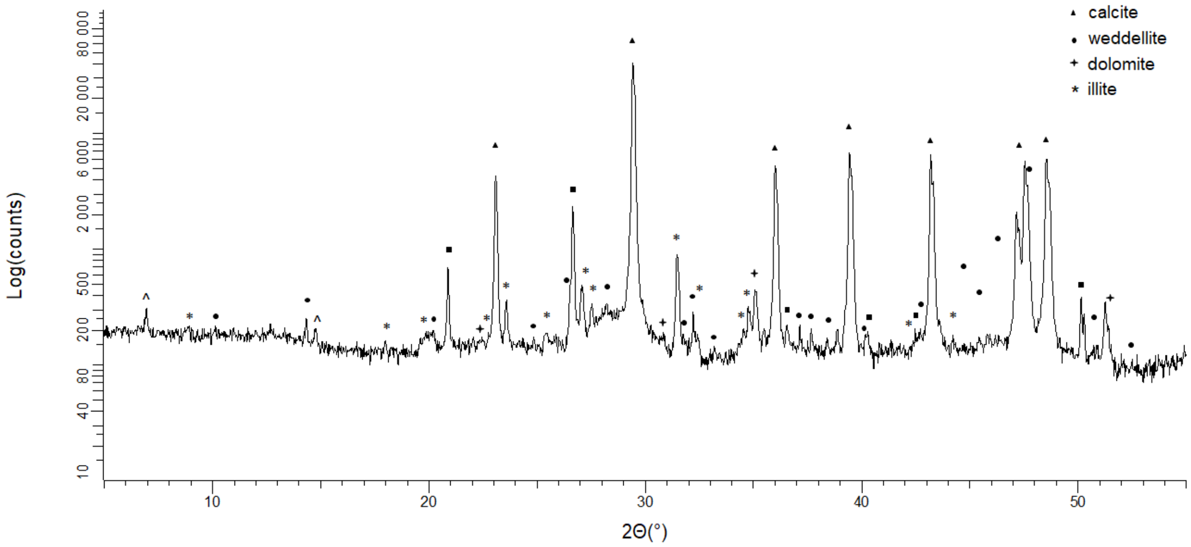

Figure S25. XRD diffractogram of a powdered sample of biomicrite limestone treated with a 12% w/w aqueous solution of compound **2**. Peaks at 7 and 14° (symbol ^) were not assigned.

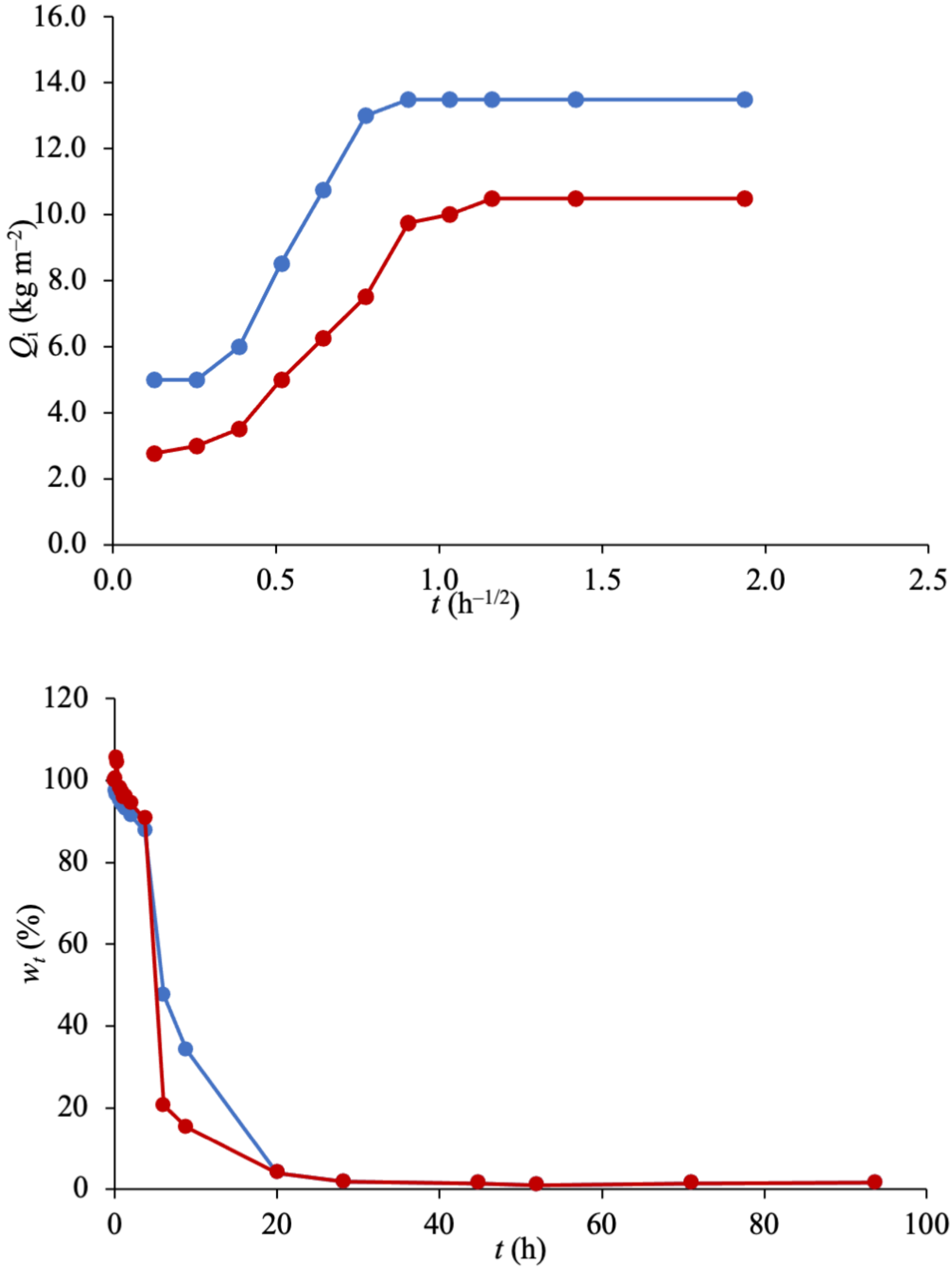

Figure S26. Water capillary absorption (top) and desorption (bottom) curves for thermally biomicritic limestone samples before (blue) and after (red) the treatment with compound **2** solution.

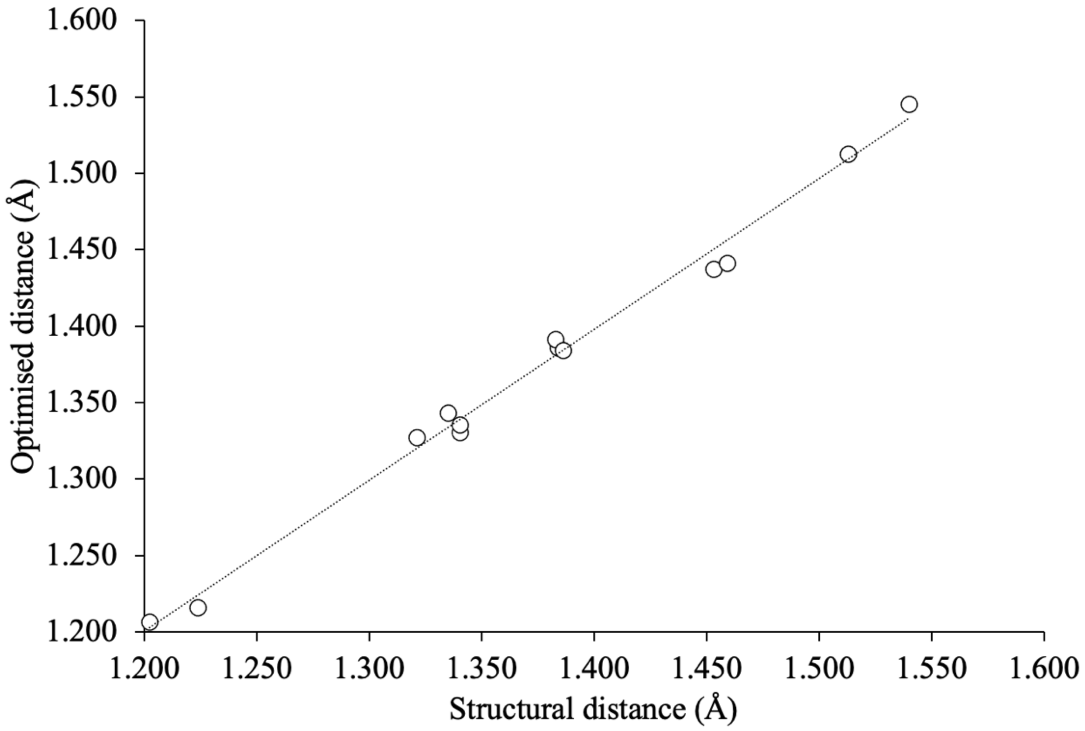

Figure S27. Optimized vs experimental bond distances for selected bond distances in compound **1** (calculations at B3LYP/def2-TZVP). Correlation coefficient $R = 0.996$.

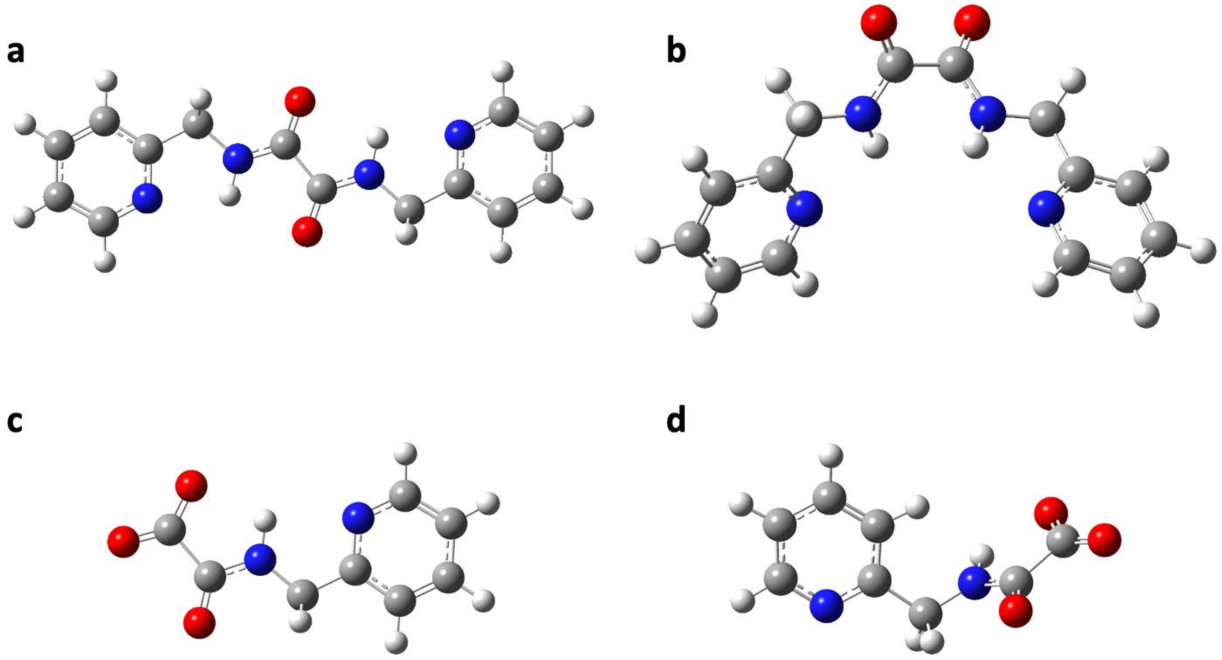

Figure S28. Representations of the B3LYP/def2-TZVP optimized structures of compound **3** in the antiperiplanar (a) and periplanar (b) configuration, and PicOxam⁻ anion with the pyridine ring coplanar (c) or rotated (d) with respect to the oxamate plane.

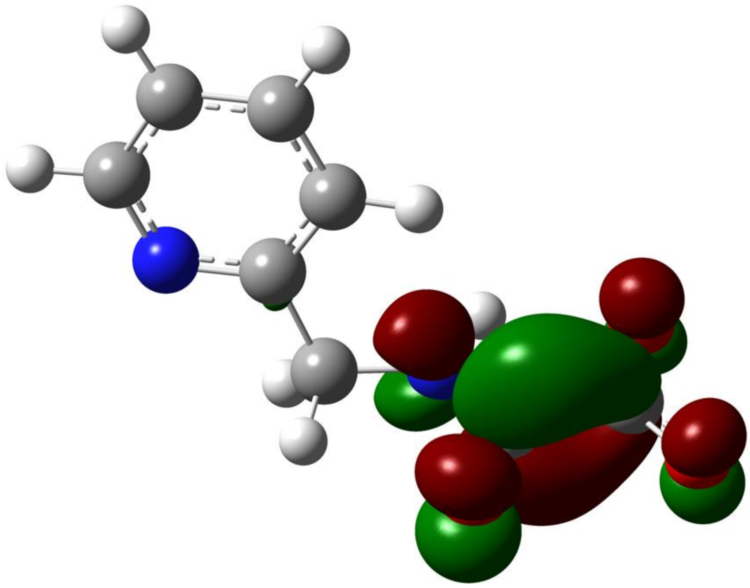

Figure S29. Isosurface of KS-LUMO+5 calculated for the PicOxam^{-} anion at B3LYP/def2-TZVP level. Cutoff value = 0.05 |e|.

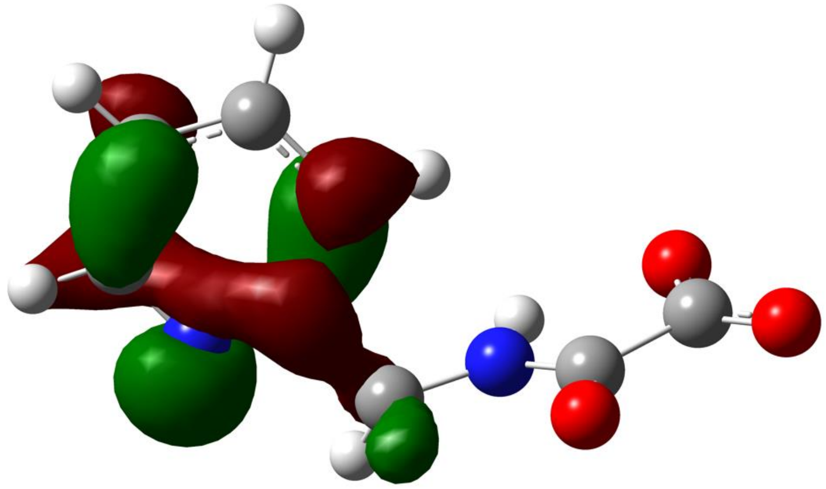

Figure S30. Isosurface of KS-HOMO–5 calculated for the PicOxam^{-} anion at B3LYP/def2-TZVP level. Cutoff value = 0.05 |e|.

Table S1. Crystallographic data and refinement parameters for **1** and **2**·1/2H_2O.

**Compound**	**1**	**2**·1/2H_2O
Formula	$C_9H_{10}N_2O_3$	$C_8H_{12}N_3O_{3.5}$
$D_{calc.}$/ g·cm^{-3}	1.410	1.382
μ/mm^{-1}	0.108	0.932
Formula Weight	194.19	206.21
Colour	colourless	colourless
Shape	(cut) plate	block
Size / mm^3	0.380×0.312×0.068	0.260×0.190×0.100
T / K	100(2)	100(2)
Crystal System	monoclinic	triclinic
Space Group	$P2_1/c$	P-1
a / Å	4.46650(10)	7.40430(10)
b / Å	10.1433(3)	11.1729(2)
c / Å	20.2049(5)	12.71920(10)
α / °	90	105.2210(10)
β / °	91.796(2)	98.0690(10)
γ / °	90	97.0870(10)
V / Å^3	914.93(4)	991.02(2)
Z	4	4
Z'	1	2
Wavelength / Å	0.71075	1.54184
Radiation type	Mo$K\alpha$	Cu$K\alpha$
Θ_{min} / °	2.247	3.662
Θ_{max} / °	27.483	68.243
Measured Refl.	39502	18194
Independent Refl.	2097	3562
Reflections with I > 2(I)	1916	3508
R_{int}	0.0547	0.0221
Parameters	131	297
Restraints	0	0
Largest Peak	0.298	0.468
Deepest Hole	-0.268	-0.203
GooF	1.036	1.131
wR_2 (all data)	0.0831	0.1141
wR_2	0.0807	0.1139
R_1 (all data)	0.0354	0.0431
R_1	0.0322	0.0428

Table S2. Selected bond lengths (Å) and angles (°) for compound **1.**

	**Length (Å)**		**Angle (°)**
C1−C2	1.540(14)	O1−C1−C2	110.97(8)
C1−O1	1.3212(12)	O2−C1−C2	123.22(9)
C1−O2	1.2025(13)	O2−C1−O1	125.80(10)
C2−N1	1.3349(13)	N1−C2−C1	112.56(8)
C2−O3	1.2239(12)	O3−C2−C1	121.73(9)
C3−C4	1.5130(14)	O3−C2−N1	125.71(10)
C3−N1	1.4591(13)	N1−C3−C4	112.31(8)
C4−C5	1.3862(14)	C5−C4−C3	121.28(9)
C4−N2	1.3401(13)	N2−C4−C3	116.19(9)
C5−C6	1.3862(15)	N2−C4−C5	122.53(9)
C6−C7	1.3828(16)	C4−C5−C6	119.23(10)
C7−C8	1.3840(15)	C7−C6−C5	118.63(10)
C8−N2	1.3406(14)	C6−C7−C8	118.49(10)
C9−O1	1.4529(13)	N2−C8−C7	123.49(10)
		C2−N1−C3	120.04(9)
		C4−N2−C8	117.63(9)
		C1−O1−C9	115.83(8)

Table S3. Selected bond lengths (Å) and angles (°) for compound **2**·1/2H_2O.

	Length (Å)		Angle (°)
O2−C1	1.2476(19)	C10−N3−C11	120.35(13)
O1−C1	1.247(2)	C4−N2−C8	117.67(14)
O4−C9	1.246(2)	C16−N4−C12	117.84(14)
O5−C9	1.2475(19)	C2−N1−C3	120.46(13)
O6−C10	1.2301(19)	O2−C1−C2	116.95(13)
O3−C2	1.2286(19)	O1−C1−O2	128.04(14)
N3−C10	1.329(2)	O1−C1−C2	114.98(13)
N3−C11	1.4526(19)	O4−C9−O5	128.17(15)
N2−C4	1.341(2)	O4−C9−C10	115.31(13)
N2−C8	1.346(2)	O5−C9−C10	116.51(13)
N4−C12	1.344(2)	O3−C2−N1	124.21(14)
N4−C16	1.343(2)	O3−C2−C1	120.93(14)
N1−C2	1.334(2)	N1−C2−C1	114.86(13)
N1−C3	1.4543(19)	O6−C10−N3	123.98(14)
C1−C2	1.552(2)	O6−C10−C9	121.10(14)
C9−C10	1.551(2)	N3−C10−C9	114.92(13)
C12−C11	1.515(2)	N4−C12−C11	115.91(14)
C12−C13	1.386(2)	N4−C12−C13	122.10(14)
C4−C3	1.519(2)	C13−C12−C11	121.97(14)
C4−C5	1.388(2)	N2−C4−C3	115.52(13)
C13−C14	1.381(2)	N2−C4−C5	122.24(15)
C5−C6	1.384(2)	C5−C4−C3	122.22(14)
C6−C7	1.385(2)	N3−C11−C12	112.64(13)
C8−C7	1.383(2)	N1−C3−C4	113.53(13)
C14−C15	1.381(2)	C14−C13−C12	119.28(15)
C15−C16	1.382(2)	C6−C5−C4	119.19(15)
		C5−C6−C7	119.30(15)
		N2−C8−C7	123.85(15)
		C13−C14−C15	119.15(15)
		C14−C15−C16	118.21(16)
		N4−C16−C15	123.41(15)
		C8−C7−C6	117.75(15)

Table S4. Hydrogen bonding interactions found in the crystal structure of **2**·1/2H_2O.

	$d_{D–H}$ (Å)	$d_{H\cdots A}$ (Å)	$d_{D\cdots A}$ (Å)	$\alpha_{D–H\cdots A}$ (°)
O7–H7···N4^{i}	0.85	1.96	2.806(2)	172(2)
O7–H7···N2	0.85	2.01	2.856(3)	171(2)
N5–H5A···O6^{iii}	0.89(2)	1.99(2)	2.824(2)	155(2)
N5–H5B···O7	0.88(3)	2.00(2)	2.861(2)	165(2)
N5–H5C···O4^{iv}	0.94(3)	1.89(3)	2.815(2)	173.5(2)
N5–H5D···O2^{v}	0.96(3)	1.82(2)	2.762(2)	170(2)
N6–H6A···O3	0.89(2)	2.07(2)	2.865(2)	149.3(2)
N6–H6B···O7^{vi}	0.87(2)	2.01(2)	2.862(2)	165(2)
N6–H6C···O5^{vii}	0.93(3)	1.83(3)	2.753(2)	171.5(2)
N6–H6D···O1^{viii}	0.97(3)	1.83(2)	2.804(2)	177(2)
N1–H1···O5^{iii}	0.88	2.09	2.915(2)	155(2)
N3^{iii}–H3^{iii}···O2	0.88	2.04	2.900(2)	164(2)

Symmetry codes: ^{i} = −1−x, 1−y, −z; ^{iii} = −x, 1−y, −z; ^{iv} = −1+x, 1+y, +z; ^{v} = −x, 2−y, 1−z; ^{vi} = 1+x, y, z; ^{vii} = x, 1+y, z; ^{viii} = 1−x, 2−y, 1−z.

Table S5. Petrographic, dynamic, structural, and colorimetric characterization of marble samples before ("intact") and after ("weathered") the weathering process.

Property	"Intact" Carrara white marble	Weathered Carrara white marble
**Petrography**		
Mineralogic composition	Calcite, muscovite	Calcite, muscovite
**Dynamics**		
t_{uts} (μm)	13.4 ± 0.1	52.4 ± 0.1
v_{uts} ($km{\cdot}s^{-1}$)	6.2 ± 0.4	1.5 ± 0.1
E_d ($MN{\cdot}m^{-2}$)	67 ± 5	4.23 ± 0.08
**Structural**		
ρ_a ($g{\cdot}cm^{-3}$)	2.68 (0.01)	2.62 (0.01)
ρ_r ($g{\cdot}cm^{-3}$)	2.714 (0.004)	2.708 (0.003)
I_c %	98.82	96.75
ϕ %	1.3 (0.2)	3.0 (0.4)
**Colorimetry**		
L^*	84.97	92.90
a^*	0.41	0.25
b^*	0.23	0.26
C	0.47	3.08
$WI_{CIELAB76}$	64.81	68.56
$YI_{ASTME313}$	0.84	6.15
$\Delta E^*_{CIE1976}$	–	8.42
$\Delta E^*_{CIE2000}$	–	5.69
**Roughness**		
Ra (μm)	2.7 (0.2)	2.6 (0.3)
Rz (μm)	25 (4)	27 (5)
Rq (μm)	3.5 (0.2)	(0.2)

Table S6. pH Values and conductivity (mS/cm) determined for the water solution of compound **2** before and after the treatment of Carrara marble and biomicrite limestone.

	**pH**	**κ (mS/cm)**
5% w/w solution	6.53 ± 0.01	8.80 ± 0.04
White marble treated with 5% w/w sol.	9.12 ± 0.01	6.50 ± 0.03
Biomicritic limestone treated with 5% w/w sol.	8.36 ± 0.01	7.25 ± 0.04
12% w/w solution	6.56 ± 0.01	28.8 ± 0.1
White marble treated with 12% w/w sol.	9.09 ± 0.01	15.12 ± 0.08
Biomicritic limestone treated with 12% w/w sol.	8.00 ± 0.01	16.52 ± 0.08

Table S7. Root-mean-squared deviations calculated for selected metric parameters optimized at DFT level and compared to the corresponding structural ones for compound **1**.[a,b,c]

	**Distances**	**Angles**
B3LYP/def2-SVP	0.336	0.243
B3LYP/def2-TZVP	0.235	0.199
B3LYP/6-311G	0.485	0.277
mPW1PW/def2-SVP	0.379	0.247
mPW1PW/def2-TZVP	0.328	0.203
mPW1PW 6-311G	0.409	0.281
PBE0/def2-SVP	0.388	0.244
PBE0/def2-TZVP	0.334	0.203
PBE0/6-311G	0.421	0.275

[a] Selected bond distances: C1–C2, C4–C5, C3–C4, C5–C6, C6–C7, C7–C8, C2–N1, N1–C3, C4–N2, C8–N2, O1–C1, O2–C1, O3–C2. [b] Selected bond angles: C2–N1–C3, C4–N2–C8, C4–C5–C6, C3–C4–C5, C5–C6–C7, C6–C7–C8, C1–C2–N1, N1–C3–C4, N2–C4–C5, C7–C8–N2, O1–C1–C2, O2–C1–C2, O3–C2–N1. [c] Numbering scheme as in Fig. S1.

Table S8. Geometry of compound **1** in orthogonal Cartesian format optimized at B3LYP/def2-TZVP DFT level.

**Center Number**	**Atomic Number**	**Coordinates (Å)**		
		**X**	**Y**	**Z**
1	6	-0.029958	0.313016	0.252485
2	6	0.078165	0.422779	1.629645
3	6	1.341269	0.302472	2.200459
4	6	2.430652	0.079884	1.375911
5	6	2.225083	-0.016205	-0.001718
6	7	1.017102	0.098585	-0.548107
7	6	3.397413	-0.256924	-0.926836
8	7	2.973462	-0.340095	-2.301656
9	6	3.847898	-0.565743	-3.296576
10	8	5.046387	-0.718852	-3.159455
11	6	3.146602	-0.611208	-4.672963
12	8	1.957968	-0.459169	-4.817062
13	8	4.012957	-0.835556	-5.652891
14	6	3.459853	-0.896342	-6.978452
15	1	4.303650	-1.083581	-7.635792
16	1	2.730227	-1.702417	-7.047847
17	1	2.975973	0.046640	-7.230617
18	1	1.987843	-0.222894	-2.499879
19	1	4.132771	0.546457	-0.808132
20	1	3.915396	-1.177683	-0.637851
21	1	-0.994017	0.399708	-0.236798
22	1	-0.800155	0.596965	2.236459
23	1	1.472303	0.381094	3.272356
24	1	3.428222	-0.019194	1.785848

Table S9. Geometry of the PicOxam⁻ anion in orthogonal Cartesian format optimized at B3LYP/def2-TZVP DFT level.

Center Number	Atomic Number	Coordinates (Å)		
		X	Y	Z
1	8	2.209388	-0.222530	1.675174
2	8	0.535709	0.273299	3.120031
3	8	0.812365	1.435900	-0.203322
4	7	-0.817778	1.595891	1.376232
5	1	-0.980321	1.296336	2.333029
6	7	-2.832367	4.599039	0.725749
7	6	1.145797	0.298892	2.017486
8	6	0.394515	1.163722	0.910794
9	6	-1.804801	3.871128	1.182233
10	6	-1.694261	2.457748	0.643360
11	1	-2.708540	2.050288	0.576784
12	1	-1.300679	2.508943	-0.377614
13	6	-0.878160	4.388445	2.089845
14	1	-0.071982	3.759252	2.440786
15	6	-1.020370	5.696965	2.524194
16	1	-0.311595	6.118307	3.227329
17	6	-2.082306	6.455677	2.046457
18	1	-2.233964	7.481028	2.359458
19	6	-2.956674	5.854971	1.150046
20	1	-3.803172	6.410955	0.755424

Table S10. Geometry of compound **3** in orthogonal Cartesian format optimized at B3LYP/def2-TZVP DFT level.

Center Number	Atomic Number	Coordinates (Å)		
		X	Y	Z
1	7	0.467455	-0.941987	0.306967
2	6	0.496760	-0.236014	1.434382
3	6	1.513311	-0.386844	2.380030
4	6	2.523493	-1.301167	2.136181
5	6	2.493026	-2.039566	0.957321
6	6	1.444741	-1.822348	0.077291
7	6	-0.619592	0.755173	1.676975
8	7	-1.560787	0.772380	0.586627
9	6	-2.626717	1.582134	0.579142
10	8	-2.918540	2.374770	1.464794
11	6	-3.486912	1.421970	-0.691973
12	8	-3.195524	0.628766	-1.577259
13	7	-4.552276	2.232465	-0.699935
14	6	-5.492746	2.250620	-1.790893
15	6	-6.594636	3.260713	-1.560469
16	6	-7.597285	3.429714	-2.517813
17	6	-8.595723	4.359390	-2.283848
18	6	-8.567555	5.094787	-1.103055
19	6	-7.533261	4.859280	-0.211254
20	7	-6.567473	3.963888	-0.431253
21	1	-4.707389	2.855496	0.083533
22	1	-5.929091	1.254923	-1.927356
23	1	-4.974494	2.477525	-2.728926
24	1	-7.469740	5.408487	0.721923
25	1	-9.327444	5.831532	-0.879690
26	1	-9.384995	4.510471	-3.009562
27	1	-7.587624	2.839876	-3.426179
28	1	-1.405562	0.149495	-0.196935
29	1	-0.198907	1.755725	1.824954
30	1	-1.133305	0.508872	2.612851
31	1	1.379086	-2.374341	-0.854092
32	1	3.261880	-2.764592	0.726412
33	1	3.323778	-1.437852	2.852629
34	1	1.505307	0.205159	3.287002
